# Podocalyxin enhances breast tumor growth and metastasis and is a target for monoclonal antibody therapy

**DOI:** 10.1186/s13058-015-0562-7

**Published:** 2015-03-27

**Authors:** Kimberly A Snyder, Michael R Hughes, Bradley Hedberg, Jill Brandon, Diana Canals Hernaez, Peter Bergqvist, Frederic Cruz, Kelvin Po, Marcia L Graves, Michelle E Turvey, Julie S Nielsen, John A Wilkins, Shaun R McColl, John S Babcook, Calvin D Roskelley, Kelly M McNagny

**Affiliations:** The Biomedical Research Centre, University of British Columbia, 2222 Health Sciences Mall, Vancouver, BC V6T 1Z3 Canada; Centre for Drug Research and Development, University of British Columbia, Vancouver, BC V6T 1Z3 Canada; Department of Cellular and Physiological Sciences, University of British Columbia, Vancouver, BC V6T 1Z3 Canada; Centre for Molecular Pathology, School of Molecular & Biological Science, The University of Adelaide, Adelaide, SA 5005 Australia; Department of Internal Medicine, Manitoba Centre for Proteomics and Systems Biology, University of Manitoba, Winnipeg, MB Canada

## Abstract

**Introduction:**

Podocalyxin (gene name *PODXL*) is a CD34-related sialomucin implicated in the regulation of cell adhesion, migration and polarity. Upregulated expression of podocalyxin is linked to poor patient survival in epithelial cancers. However, it is not known if podocalyxin has a functional role in tumor progression.

**Methods:**

We silenced podocalyxin expression in the aggressive basal-like human (MDA-MB-231) and mouse (4T1) breast cancer cell lines and also overexpressed podocalyxin in the more benign human breast cancer cell line, MCF7. We evaluated how podocalyxin affects tumorsphere formation *in vitro* and compared the ability of podocalyxin-deficient and podocalyxin-replete cell lines to form tumors and metastasize using xenogenic or syngeneic transplant models in mice. Finally, in an effort to develop therapeutic treatments for systemic cancers, we generated a series of antihuman podocalyxin antibodies and screened these for their ability to inhibit tumor progression in xenografted mice*.*

**Results:**

Although deletion of podocalyxin does not alter gross cell morphology and growth under standard (adherent) culture conditions, expression of *PODXL* is required for efficient formation of tumorspheres *in vitro.* Correspondingly, silencing podocalyxin resulted in attenuated primary tumor growth and invasiveness in mice and severely impaired the formation of distant metastases. Likewise, in competitive tumor engraftment assays where we injected a 50:50 mixture of control and shPODXL (short-hairpin RNA targeting *PODXL*)-expressing cells, we found that podocalyxin-deficient cells exhibited a striking decrease in the ability to form clonal tumors in the lung, liver and bone marrow. Finally, to validate podocalyxin as a viable target for immunotherapy, we screened a series of novel antihuman podocalyxin antibodies for their ability to inhibit tumor progression *in vivo*. One of these antibodies, PODOC1, potently blocked tumor growth and metastasis.

**Conclusions:**

We show that podocalyxin plays a key role in the formation of primary tumors and distant tumor metastasis. In addition, we validate podocalyxin as potential target for monoclonal antibody therapy to inhibit primary tumor growth and systemic dissemination.

**Electronic supplementary material:**

The online version of this article (doi:10.1186/s13058-015-0562-7) contains supplementary material, which is available to authorized users.

## Introduction

Although most human cancers begin as primary focal lesions, metastasis of these primary tumors to distant sites heralds advanced stage disease, poor prognosis and eventual patient death [1]. For this reason, biomarkers that identify tumors likely to metastasize, and the generation of therapeutics that can inhibit metastasis, are key to improving patient survival. Although adjuvant therapies have been developed for several types of breast tumors, triple-negative breast cancers (estrogen receptor (ER)-, progesterone receptor (PgR)- and human epidermal growth factor receptor 2 (HER2)-negative) are particularly challenging to treat because of their highly aggressive nature and a lack of well-defined therapeutic targets on these cells [2].

Podocalyxin (also known as PCLP1, MEP21, gp135, TRA-1-60, TRA-1-81 and GCTM2) is a CD34-related sialomucin and a well-known marker of embryonic stem cells, embryonal carcinomas, neoplastic hematopoietic cells [3-6] and a variety of normal cells during embryonic development, where it plays a key role in tissue morphogenesis [7-9]. We previously showed that podocalyxin (gene name *PODXL*) is upregulated on a subset of primary breast tumors and is an independent predictor of progression, metastasis and poor outcome [10]. Subsequent studies have confirmed podocalyxin as a prognostic indicator of poor outcome in a variety of malignancies, including ovarian, prostate, renal, pancreatic, thyroid, glioblastoma, astrocytoma, colorectal and bladder cancers [10-18]. Ectopic expression of *PODXL* enhances tumor aggressiveness *in vitro*. MCF7 breast tumor cells engineered to express high levels of murine podocalyxin (MCF7^Podxl^) exhibit increased migration *in vitro*, altered morphogenesis and disrupted cell–cell and cell–matrix contacts [10,19,20]. In addition, podocalyxin has been shown to play a role in the control of cell migration and the expression of matrix metalloproteinases MMP1 and MMP9 [17,21]. Collectively, these studies establish a correlation between podocalyxin expression, tumor aggressiveness and poor outcome (reviewed by McNagny *et al*. [22]). However, the functional significance of podocalyxin expression by primary tumors and its influence on metastatic progression *in vivo* have yet to be thoroughly evaluated. In the present study, we have addressed this issue by silencing podocalyxin expression in the highly aggressive triple-negative basal-like human breast cancer cell line, MDA-MB-231, or overexpressing it in a well-differentiated, ER-positive and PgR-positive, luminal-like human breast cancer cell line, MCF7 [23]. We found that podocalyxin is required for efficient tumorsphere formation in both MCF7 and MDA-MB-231 cells. Moreover, suppression of *PODXL* in MDA-MB-231 cells profoundly impairs formation of primary tumors and secondary metastasis in xenografted mice. We recapitulated this finding in an immunocompetent mouse tumor model by silencing podocalyxin expression in 4T1 cells (a mouse mammary tumor line) and engrafting these cells in syngeneic BALB/c mice. Finally, we developed a novel podocalyxin-specific monoclonal antibody (mAb) that delays xenografted tumor formation and metastatic disease in mice. These data validate podocalyxin as a regulator of tumor progression and a novel therapeutic target.

## Methods

### Cell culture

MDA-MB-231, MCF7 and 4T1 cells (American Type Culture Collection, Manassas, VA, USA) were grown as monolayers on tissue culture-treated plastic plates. All cell lines were maintained in low passage (<15). Both MDA-MB-231 and MCF7 human breast tumor cell lines were cultured in Dulbecco’s modified Eagle’s medium (DMEM) supplemented with 10% fetal bovine serum (FBS), penicillin and streptomycin. 4T1 BALB/c mouse-derived mammary tumor cells were cultured in DMEM supplemented with 10% FBS, 2 mM glutamine, nonessential amino acids, penicillin and streptomycin. All cell lines were cultured in a humidified 5% CO_2_ incubator at 37°C.

### Transduction

MDA-MB-231 cells were labeled with green fluorescent protein (GFP) or red fluorescent protein (RFP) using retroviral vectors pLNCX2-GFP or pLNCX2-RFP, respectively (Clontech Laboratories, Mountain View, CA, USA). Human *PODXL* was silenced in MDA-MB-231 cells by lentiviral infection using pLKO.1 containing either a scrambled short-hairpin RNA (shRNA) (shCTRL) or a *PODXL*-targeting shRNA (RHS3979-9848792, shPODXL) as recommended by the manufacturer (Dharmacon, Lafayette, CO, USA). All cell lines were derived from pooled cultures of infected cells. Cells were cultured under continuous drug selection with puromycin (4 μg/ml; Invitrogen, Carlsbad, CA, USA) and G418 (1 mg/ml; Calbiochem, San Diego, CA, USA). *PODXL*-transfected MCF7 cells were described previously [10,19]. Cells were cultured under continuous selection with gentamicin (50 μg/ml; Calbiochem).

Predicted shRNA sequences to target murine 4T1 *Podxl* were identified using pSicoOligomaker v1.5 freeware (http://web.mit.edu/jacks-lab/protocols/pSico.html). Three individual shRNA oligomers were each cloned into the *HpaI* and *XhoI* sites of the pLL3.7 lentiviral vector. Firefly luciferase-expressing 4T1 (4T1-luc) cells were maintained under selection in G418 (400 μg/ml; Calbiochem). To produce lentiviral particles, 293T cells were cotransfected with 10 μg of pLL3.7 and the appropriate packaging plasmids (3.5 μg of pVSVg, 3.5 μg of pRSV-Rev, 6.5 μg of pMDLgag/pol) by calcium phosphate transfection. Lentivirus-containing media were collected 36 hours post-transfection and transferred to subconfluent 4T1 cells seeded 1 day earlier. The virus-containing medium was replaced with regular growth media after 48 hours and incubated for an additional 48 hours. The cells were then harvested for analysis of expression of mouse podocalyxin RNA and protein. 4T1 cells with the most efficient knockdown were used for all studies and cultured with gentamicin (50 μg/ml; Calbiochem).

### Quantitative RT-PCR

RNA isolation was performed using TRIzol reagent (Life Technologies, Carlsbad, CA, USA) according to the manufacturer’s instructions. Total RNA (2 μg) was reverse-transcribed using a high-capacity cDNA reverse transcription kit (Life Technologies). Real-time quantitative PCR was performed using a SYBR FAST qPCR kit (Kapa Biosystems, Wilmington, MA, USA). The *PODXL*-specific primers used were 5′-CTCACCGGGGACTACAACC-3′ (forward) and 5′-GCCTCCTCTAGCCACGGTA-3′ (reverse). Relative expression of *PODXL* was determined relative to *GAPDH* in each reaction.

### Tumorsphere assay

MDA-MB-231 and MCF7 cells were harvested, and spheres were cultured in MammoCult™ medium (StemCell Technologies, Vancouver, BC, Canada). After 7 days, tumorspheres larger than 60 μm in diameter were counted manually using a counting grid. Tumorsphere-forming efficiency was calculated as follows: number of tumorspheres divided by number of cells initially plated times 100.

### *In vivo* tumor growth and lung metastasis

For *in vivo* experiments, we used 6- to 12-week-old female nonobese diabetic severe combined immunodeficiency, interleukin 2 receptor gamma chain deficient, NOD.Cg-*Prkdc*^scid^*Il2rg*^*t*m1Wjl^/SzJ (NSG) mice or BALB/cJ mice (The Jackson Laboratory, Bar Harbor, ME, USA). Animals were maintained in a specific pathogen-free facility at the University of British Columbia (UBC) Biomedical Research Centre. All experiments were conducted with approval of the UBC Animal Care Committee.

Primary tumor development was examined following subcutaneous (s.c.) injection of MDA-MB-231 cells (1 × 10^6^) prepared in BD Matrigel™ (BD Biosciences, San Jose, CA, USA) into the right hind flank of NSG mice. Tumor growth was measured using manual calipers, and the tumor volume was estimated using the following formula: length *times* width^2^ divided by 2. Final tumor masses were measured after excision and the tumors were retained for histochemical analyses. Flow cytometry was performed on lung digests to enumerate tumor cells based on detection of GFP or RFP fluorescence.

### Competitive experimental metastases

To examine experimental metastasis, a 50:50 mixture of 0.5 to 2.0 × 10^5^ shCTRL^RFP^ (or shCTRL^GFP^) and shPODXL^GFP^ (or shPODXL^RFP^) MDA-MB-231 cells were resuspended in 100 μl of Hanks’ balanced salt solution and injected into the tail vein of NSG mice. At day 3, 7 or 14 postinjection, mice were killed using 2,2,2-tribromoethanol (Avertin; Sigma-Aldrich, St Louis, MO, USA), then perfused through the right ventricle with 10 ml of phosphate-buffered saline (PBS) containing 2 mM ethylenediaminetetraacetic acid (EDTA), and the lungs (and, in some experiments, liver, femurs and tibias) were removed. Lungs were digested in collagenase/dispase solution as described elsewhere [24], and GFP-positive or RFP-positive tumor cells were detected by flow cytometry. At 6 weeks postinjection, NSG mice were killed and perfused as described above, but tumor nodules on the surface of lungs and livers were manually counted using a Leica Fluo™ dissecting microscope (Leica Microsystems, Buffalo Grove, IL, USA) and QImaging™ software (QImaging, Surrey, BC, Canada). In addition, lung, liver and bone marrow cells were prepared as described previously and analyzed by flow cytometry.

### Flow cytometry

Staining was performed with PBS containing 2% FBS, 2 mM EDTA and 0.05% sodium azide. MDA-MB-231 cells were stained with a primary antibody (Ab) against podocalyxin (goat antihuman podocalyxin antibody (anti-PODO Ab); R&D Systems, Minneapolis, MN, USA) or a goat immunoglobulin G (IgG) isotype control (Iso) and followed with a chicken anti-goat Alexa Fluor (AF) 647–coupled secondary Ab (Molecular Probes, Eugene, OR, USA) for 30 minutes at 4°C and analyzed using a BD LSR II flow cytometer (BD Biosciences). Murine 4T1-luc cells were labeled with allophycocyanin-conjugated rat anti-mouse podocalyxin Ab (R&D Systems) and analyzed by flow cytometry. Rat IgG2b was used as an isotype control.

### Experimental lung metastasis

A total of 1 × 10^5^ vector control (VC) or shPODXL 4T1-luc cells were injected intravenously (i.v.) into the lateral tail vein BALB/c mice. Lungs were perfused and excised as described above. Tumor burden was assessed by counting nodules visible on the surface of the lungs using a dissecting microscope and then corroborated by performing a luciferase assay of homogenized lung tissue.

### Luciferase enzymatic assay

Total luciferase activity was assayed from lungs harvested from BALB/c mice injected i.v. with 4T1-luc cells. Lungs were homogenized in cell lysis buffer (Promega, Madison, WI, USA). Protein concentration was determined using a Thermo Scientific Pierce bicinchoninic acid protein assay kit (Pierce Biotechnology, Rockford, IL, USA). The Dual-Luciferase Reporter Assay System (Promega) was used to detect luciferase activity. In these experiments, 20 μl of sample supernatant was mixed with 50 μl of luciferase assay reagent, and luciferase activity was quantified using a SpectraMax L microplate reader (Molecular Devices, Sunnyvale, CA, USA). The results are reported as relative light units.

### Therapeutic antibody production

New Zealand White rabbits were immunized with A-172 glioblastoma cells that express high levels of tumor-glycosylated human podocalyxin on their cell surface. Rabbit mAbs were rescued as previously described [25]. Briefly, individual B-cell clones were isolated from animals whose sera recognized MDA-MB-231 cell–expressed podocalyxin extracellular domain by enzyme-linked immunosorbent assay. Next, supernatants were screened against MDA-MB-231 and human embryonic kidney 293 (HEK293) cells with and without podocalyxin on their surface (both cell lines express endogenous podocalyxin) to ensure immunoreactivity to the native protein and minimal nonspecific binding to *PODXL*-deficient cells. Finally, supernatants were also screened using Chinese hamster ovary (CHO) cells expressing podocalyxin and CD34 to ensure podocalyxin specificity. By comparing binding selectivity for podocalyxin expressed on tumor and normal cells, B-cell clones that produced Abs with favorable binding profiles to tumor cells were selected for cloning, scale-up production and *in vivo* screening.

### Preclinical mouse model to assess anti-podocalyxin therapeutic antibody efficacy

Candidate anti-PODO Abs were selected based on the level of binding to known podocalyxin-expressing tumor cell lines (Table 1). MDA-MB-231^RFP^ tumor cells (1 × 10^6^ cells) were incubated with 25 μg of anti-PODO Ab (PODOC1 through PODOC8) or Iso Ab (anti-ovalbumin) at room temperature for 30 minutes. Prior to injection, the tumor cell/Ab mixture was diluted in Matrigel™ and injected s.c. into the flank region of NSG mice. For systemic therapy, beginning on day 14 after tumor injection, mice were administered 100 μg of Ab (4.5 mg/kg) by intraperitoneal (i.p.) injection twice weekly. Tumor dimensions were measured every 3 days until the mice were killed on day 27.

**Table 1:** **Binding selectivity (geometric mean) of candidate podocalyxin antibodies compared with isotype control** ^**a**^

**mAb name**	**HUVEC**	**MCF7**	**MDA-MB-231**	**T47D**	**CAOV-3**	**OVCAR-3**	**OVCAR-10**	**A-172**	**HEK293**
Isotype	489	657	477	697	767	629	653	644	446
PODOC1	44,611	60,805	91,909	1,498	48,817	16,502	2,834	169,600	28,225
PODOC2	3,231	1,962	26,513	2,036	37,683	9,189	645	49,862	459
PODOC3	2,103	9,399	2,939	818	10,472	8,355	1,803	181,211	3,869
PODOC4	113,144	15,161	143,135	34,559	125,223	29,862	564	333,980	2,679
PODOC5	40,490	25,055	80,312	1,853	31,657	9,824	1,756	172,233	27,144
PODOC6	994	1,239	4,744	2,685	1,670	1,350	1,165	2,760	485
PODOC7	21,700	22,330	57,626	1,010	37,668	11,058	2,539	278,032	13,886
PODOC8	23,207	45,707	1,950	566	52,200	8,805	2,731	263,842	5,056

^a^HEK293, Human embryonic kidney 293 cells; HUVEC, Human umbilical vascular endothelial cells; mAb, Monoclonal antibody; PODOC, Podocalyxin antibody.

### Histological analysis

Formalin-fixed, paraffin-embedded tumor specimens were serially sectioned. Representative sections were deparaffinized and stained with hematoxylin and eosin (H&E) or Ki-67 Ab (1:700; Thermo Scientific, Waltham, MA, USA) followed by donkey anti-rabbit AF488 secondary Ab (1:1,000; Invitrogen). ProLong Gold Antifade mounting compound with 4′,6-diamidino-2-phenylindole dihydrochloride (DAPI) nuclear stain (Life Technologies) was used to mount slides. H&E-stained sections were examined qualitatively for evidence of muscular invasion and tumor border integrity.

### Statistical analysis

Data are expressed as the mean ± standard error of the mean (SEM) unless indicated otherwise. A Student’s *t*-test was conducted for evaluation of statistical significance. Data generated from time-dependent studies were analyzed by two-way analysis of variance. *P* < 0.05 was considered to be statistically significant. All data presented in the figures are representative of at least two independent experiments.

## Results

### Podocalyxin promotes tumorsphere formation *in vitro*

**Figure 1:**
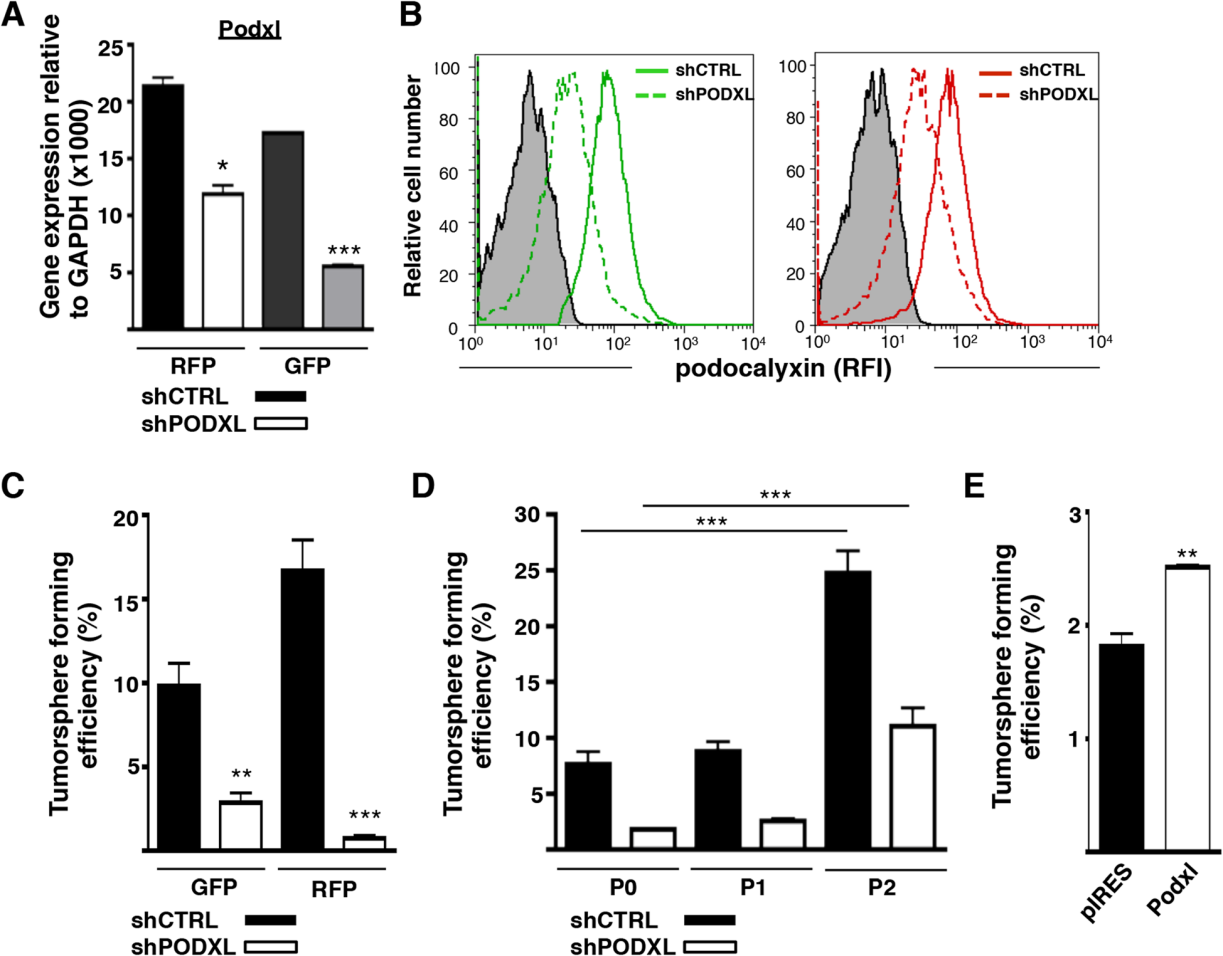
**Podocalyxin promotes tumorsphere formation. (A)** *PODXL* gene expression relative to *GAPDH* in scrambled shRNA (shCTRL) and shRNA targeting *PODXL* (shPODXL) MDA-MB-231 cells as determined by quantitative PCR (n = 3; *P < 0.05; ***P < 0.001). **(B)** Podocalyxin expression on shCTRL^GFP^ cells (green fluorescent protein (GFP); solid green) and shPODXL^GFP^ (dashed green) MDA-MB-231 cells (left histogram) and shCTRL^RFP^ (red fluorescent protein (RFP); solid red) and shPODXL^RFP^ (dotted red) MDA-MB-231 cells (right histogram) relative to isotype control (shaded). RFI, Relative fluorescence intensity. **(C)** Tumorsphere-forming efficiency of shCTRL (solid bar) and shPODXL (open bar) MDA-MB-231 cells cultured for 7 days under anchorage-independent conditions (n = 3; **P < 0.01, ***P < 0.001). **(D)** Self-renewal capacity of shCTRL^RFP^ (solid bars) and shPODXL^RFP^ (open bars) MDA-MB-231 tumorspheres assessed by serial passaging (P) (n = 3; ***P < 0.001 by one-way analysis of variance). **(E)** MCF7^pIRES^ control (solid bar) and MCF7^Podxl^ (open bar) cells were cultured under anchorage-independent conditions for 7 days to assess tumorsphere-forming efficiency (n = 3; **P < 0.01). Figures are representative of three experiments with similar results. Error bars indicate standard error of the mean.

To examine the role of podocalyxin in tumor progression, we silenced expression in MDA-MB-231 human breast cancer cells using a shRNA-containing lentivirus (shPODXL). RFP- and GFP-labeled MDA-MB-231 cells were used as a method for subsequent tracking of knockdown cells *in vivo*. Gene expression analyses confirmed attenuated *PODXL* expression in shPODXL cells (Figure 1A). In addition, flow cytometric analyses confirmed that cell surface podocalyxin expression is efficiently reduced in shPODXL^GFP^ cells (threefold decrease) compared with scrambled shRNA-infected controls (shCTRL^GFP^; Figure 1B, left panel). Likewise, podocalyxin expression in shPODXL^RFP^ cells was reduced by approximately twofold compared with shCTRL^RFP^ control (Figure 1B, right panel). Similarly, total levels of podocalyxin protein were reduced in shPODXL cells compared with control as shown by Western blotting (Additional file 1).

Although proliferation of MDA-MB-231 cells in monolayer culture was unaffected by silencing *PODXL* (Additional file 2), the frequency of tumorsphere-forming cells in three-dimensional assays was reduced by more than threefold in shPODXL cultures (Figure 1C). The comparable size (Additional file 3A, B) and morphology of control and shPODXL tumorspheres (Additional file 3B) suggests podocalyxin expression alters the frequency of tumorsphere-initiating cells rather than qualitatively affecting sphere formation *per se*. Serial passage of tumorsphere cultures is known to improve the efficiency of subsequent sphere formation [26,27], and both the shCTRL and shPODXL populations exhibited an approximately threefold increase in the frequency of sphere-initiating cells over three passages (Figure 1D). To further confirm that podocalyxin has a causal role in promoting tumorsphere formation *in vitro,* we overexpressed *PODXL* in MCF7 cells (MCF7^Podxl^) (Additional file 3C), a luminal-like human breast cancer cell line that expresses very low levels of endogenous podocalyxin [10,19]. MCF7^Podxl^ cells (Additional file 3C) exhibited a 30% increase in sphere-forming efficiency (Figure 1E). The results of these gain- and loss-of-function experiments suggest that podocalyxin expression increases the frequency of tumorsphere-forming cells in these cell lines. Because formation of tumorspheres in suspension culture provides an estimate of the frequency of tumor-initiating cells (TICs) [28-30], the observation that *PODXL* knockdown dampens tumorsphere formation is consistent with the notion that podocalyxin plays a role in TIC maintenance.

### Podocalyxin promotes primary tumor formation and metastasis

**Figure 2:**
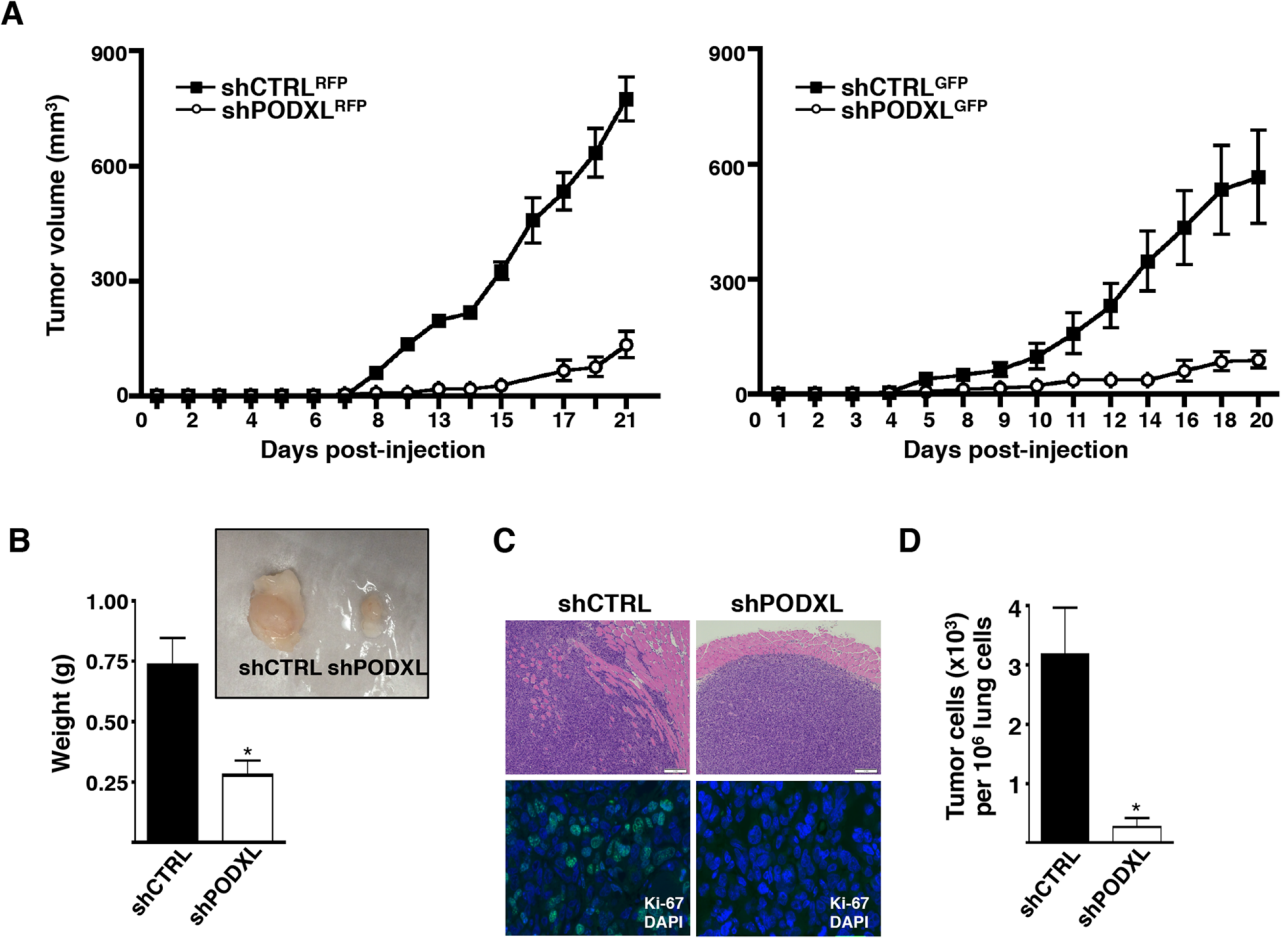
**Podocalyxin promotes primary tumor formation, local invasion and metastasis. (A)** Growth curve of subcutaneous (s.c.) flank tumors initiated in NSG mice by scrambled shRNA control (shCTRL^RFP^; closed squares) or shRNA targeting *PODXL* (shPODXL^RFP^; open circles) MDA-MB-231 cells (left; n = 5 for shCTRL^RFP^ group and n = 6 for shPODXL^RFP^ group; growth curves are significantly different with *P* < 0.001). Similarly, we compared s.c. tumor growth of shCTRL^GFP^ with shPODXL^GFP^ MDA-MB-231 cells in NSG mice (right; n = 6; growth curves are significantly different with *P* < 0.05). GFP, Green fluorescent protein; RFP, Red fluorescent protein. The final volume of the excised tumors was measured on the dates mice were killed (left; day 21, right; day 20). **(B)** Wet weight (g) of pooled shCTRL (solid bar) and shPODXL (open bar) tumors weighed immediately after excision (n = 11 for shCTRL group and n = 5 for shPODXL group; *P < 0.05). Representative photograph of excised shCTRL and shPODXL tumors (inset). **(C)** Representative H&E–stained images of shCTRL (upper left) and shPODXL (upper right) primary tumor sections (scale bar = 1 mm). Representative immunofluorescence images of shCTRL (lower left) and shPODXL (lower right) primary tumor sections showing Ki-67-positive cells (green). DAPI stain was used as a nuclear marker (blue). **(D)** The number of tumor cells detected in the lungs 20 or 21 days after subcutaneous injection. Data shown are the number of fluorescent tumor cells (×10^3^) per 10^6^ events detected by flow cytometry (n = 11 for shCTRL group and n = 5 for shPODXL group; *P < 0.05). The data shown are representative of three independent experiments.

To examine the functional significance of podocalyxin expression *in vivo*, immunocompromised mice (NSG strain) were given s.c. injections of shCTRL or shPODXL MDA-MB-231 cells into the flank. Palpable solid tumors were detected 8 days after injection of shCTRL MDA-MB-231 cells, and these rapidly increased in volume over time. In comparison, growth of shPODXL MDA-MB-231 tumors was significantly attenuated. Tumors derived from shPODXL^RFP^ cells were less than 250 mm^3^ in volume by day 21, whereas shCTRL^RFP^ tumors reached sizes greater than 750 mm^3^ (Figure 2A, left). Similar results were obtained in independent experiments using shCTRL^GFP^ and shPODXL^GFP^ MDA-MB-231 cells (Figure 2A, right). At the time the mice were killed, shCTRL tumors were 2.6-fold greater in mass than shPODXL tumors (Figure 2B). In addition, shPODXL tumors appeared to be more encapsulated, with little to no evidence of invasion into the surrounding skeletal muscle. In contrast, shCTRL tumors were highly invasive (Figure 2C, upper panel). Ki-67 expression was used to assess the level of proliferation of shCTRL and shPODXL tumor cells, and we observed a significant increase in cell proliferation (increased number of Ki-67-stained cells) in shCTRL tumors compared with shPODXL tumors (Figure 2C, lower panel), showing that 37.6 ± 3.9% of shCTRL tumor cells were undergoing active proliferation, whereas no Ki-67-positive cells were detectable in shPODXL tumors. We also examined the lungs of these mice for signs of metastases 21 days after s.c. injection. Although no visible tumor nodules were observed, tumor cells were readily detectable by flow cytometry in single-cell suspensions from the lungs. Intriguingly, there was a 12-fold decrease in the frequency of metastatic cells in the lungs of mice with established shPODXL-derived primary tumors compared with mice with shCTRL-derived primary tumors (Figure 2D). We conclude that expression of podocalyxin enhances both primary tumor growth and metastasis *in vivo*.

### Podocalyxin enhances the metastatic potential of breast cancer cells

**Figure 3:**
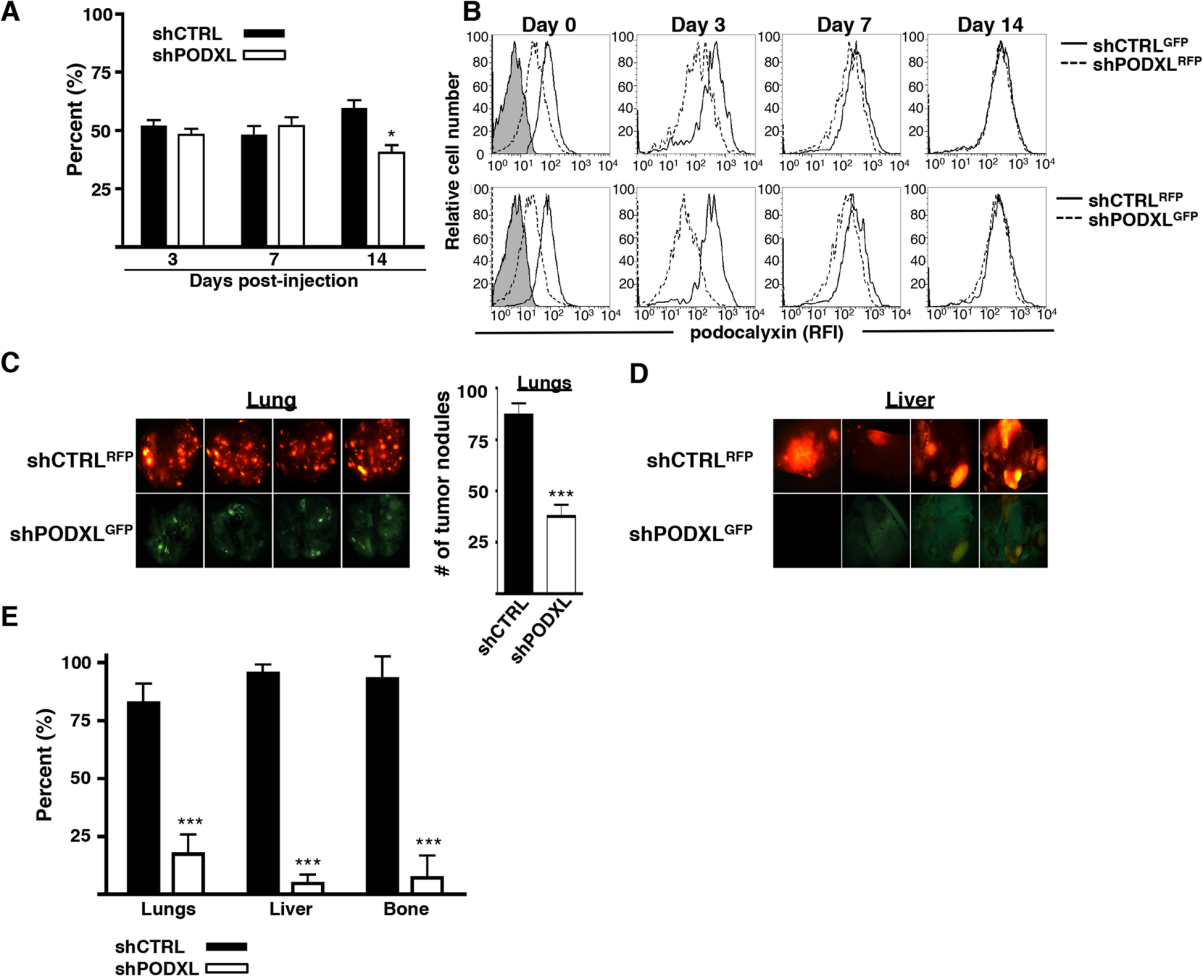
**Podocalyxin increases the metastatic burden in lungs, liver and bone marrow, but not initial lung seeding. (A)** A 50:50 mixture of scrambled shRNA control (shCTRL) and shRNA targeting *PODXL* (shPODXL) MDA-MB-231 cells were injected into the tail vein of NSG mice in a competitive experimental metastasis model. Mice were killed 3, 7 or 14 days post-injection. Presented is the relative frequency of shCTRL and shPODXL cells detected in the lungs by flow cytometry (as a % of total tumor cells) (n = 6; *P < 0.05). **(B)** Flow cytometry was performed using an antibody to detect podocalyxin expression on tumor cells in lungs isolated from NSG mice 3, 7 and 14 days post-injection. Histograms displaying the levels of (1) surface podocalyxin expression in shCTRL^GFP^ cells (solid line) compared with shPODXL^RFP^ (dashed line) cells from day 0 to day 14 (upper) and (2) surface podocalyxin expression in shCTRL^RFP^ (solid line) cells compared with shPODXL^GFP^ (dashed line) cells from day 0 to day 14 (lower). The shaded day 0 histograms represent the isotype control. RFI, Relative fluorescence intensity. **(C)** Representative fluorescence images of lungs showing shCTRL^RFP^ tumor nodules (upper left) and shPODXL^GFP^ tumor nodules (lower left) 6 weeks post-injection. The number of fluorescent tumor nodules on the lung surface was manually counted (right; n = 5; ***P < 0.001). **(D)** Representative fluorescence images of livers showing shCTRL^RFP^ tumor nodules (upper) and shPODXL^GFP^ tumor nodules (lower) (n = 5) 6 weeks post-injection. **(E)** Relative frequency of shCTRL and shPODXL tumor cells within the lungs, liver and bone marrow as determined by flow cytometry 6 weeks post-injection (n = 10; ***P < 0.001). The data shown are representative of three independent experiments. All data shown were pooled from mice injected with shCTRL^RFP^ versus shPODXL^GFP^ or shCTRL^GFP^ versus shPODXL^RFP^ run in tandem experiments.

To directly examine the functional significance of podocalyxin expression on lung-colonizing tumor cell frequency and metastatic behavior, we used an *in vivo* competitive assay (experimental lung metastasis). A 50:50 mixture of shPODXL and shCTRL MDA-MB-231 cells was injected into the tail vein of NSG mice, and relative tumor burden was evaluated at various time points over the next 6 weeks by tracking GFP or RFP expression (that is, tumor cells), respectively. Somewhat surprisingly, there was no significant difference in the ratio of shPODXL to shCTRL cells recovered from the lung at days 3 and 7 postinjection. However, we observed a slight but significant reduction in the proportion of shPODXL cells colonizing the lung by day 14 (Figure 3A). Recovered tumor cells were evaluated for podocalyxin expression 3, 7 and 14 days after injection into the mice*.* Strikingly, all shPODXL cells began to reexpress podocalyxin within 7 days of injection (likely owing to the lack of drug selection required to maintain shRNA expression). By day 14, shPODXL cells expressed podocalyxin protein at levels similar to those of shCTRL tumors (Figure 3B). A similar result was obtained with primary solid tumors seeded s.c. in the flank (Additional file 4). Nevertheless, at 6 weeks post i.v. injection, there was a 2.3-fold reduction in shPODXL cell-derived tumor nodules on the lungs compared with competing shCTRL cell-derived tumors (Figure 3C). In addition, large, cancerous lesions were visible in the liver of some mice, and, invariably, all visible liver tumors were derived from shCTRL cells as determined by fluorescence microscopy (Figure 3D). Subsequent flow cytometric analyses of lung, liver and bone marrow single-cell suspensions also revealed a dramatic under-representation of shPODXL MDA-MB-231 cells colonizing these tissues (Figure 3E). Thus, although podocalyxin expression on MDA-MB-231 cells does not alter initial “seeding” of the lung at early time points (up to 14 days), expression of podocalyxin greatly enhances subsequent establishment of clonal tumors in the lung and other organs. The similar size of the shCTRL- and shPODXL-derived lung nodules in these experimental assays (Additional file 5A) suggests that a difference in proliferation alone cannot account for the difference in the frequency of tumor nodules we observed. The higher frequency of shCTRL cells in the lungs correlates (Figure 3C) with the number of tumor nodules observed, rather than the size of the nodules.

**Figure 4:**
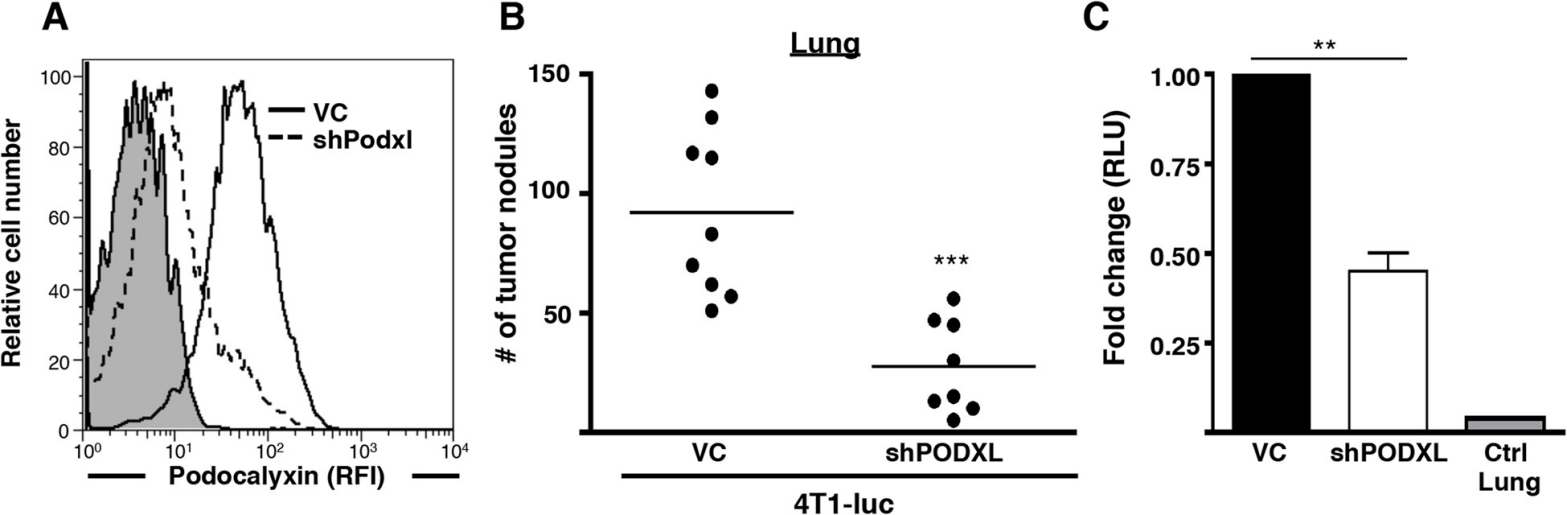
**Podocalyxin enhances metastasis of 4T1 mammary tumor cells in a syngeneic mouse model. (A)** Vector control (VC, black) and shRNA targeting *PODXL* (shPODXL; dashed line) luciferase-expressing 4T1 (4T1-luc) murine mammary tumor cells were labeled with a conjugated rat anti-mouse podocalyxin antibody and analyzed by flow cytometry to detect surface expression of podocalyxin. Rat igG_2B_ was used as an isotype control (shaded area). Data presented as histograms showing podocalyxin expression on VC cells compared with shPODXL 4T1-luc cells. RFI, Relative fluorescence intensity. **(B)** VC or shPODXL 4T1-luc cells were injected intravenously into BALB/c mice. Mice were killed after 14 days, and visible tumor nodules on the lung were manually counted using a dissecting microscope (n = 9 and n = 8, respectively; ***P < 0.001). **(C)** Luciferase expression in the lungs from noninjected, VC and shPODXL 4T1-luc-injected mice was measured using a luminometer (**P < 0.01). The data shown are representative of two independent experiments. RLU, Relative light units.

To further corroborate these findings and evaluate the functional significance of podocalyxin on tumors in an immunocompetent host, we used a syngeneic model of tumor growth and metastasis. Using shRNA, we silenced mouse podocalyxin surface protein expression by 6.3-fold in 4T1-luc mouse mammary tumor cells (BALB/c mouse-derived) (Figure 4A). Similar to our results with MDA-MB-231 cells, loss of podocalyxin expression in 4T1 cells also impaired the metastatic potential of these cells in an experimental model of lung metastasis using immunocompetent BALB/c mice (greater than threefold reduction), as shown by manual counts of tumor nodules on the lungs (Figure 4B) and by luciferase assays of lung homogenates (Figure 4C). As in the MDA-MB-231 experimental metastasis assay, the frequency of tumor nodules observed in BALB/c recipients (rather than the size of the tumor nodules) (Figure 4B and Additional file 5B) was affected by silencing *Podxl* expression in 4T1 cells. Again, the total tumor cell numbers in the lung (as measured by luciferase assay (Figure 4C)) was proportional to the frequency of tumor nodules. Thus, in both murine and human breast cancer cell lines, podocalyxin expression enhances experimental metastatic disease.

### A novel podocalyxin-specific antibody prevents primary tumor growth *in vivo*

The finding that podocalyxin expression is capable of driving breast tumor progression encouraged us to evaluate the possibility that mAbs targeting the extracellular domain of podocalyxin would prove efficacious in delaying tumor growth and metastasis. Using podocalyxin-expressing A-172 glioblastoma cells as an immunogen, we generated a novel panel of anti-human podocalyxin mAbs that exhibit preferential binding to podocalyxin expressed on human tumor cells. Of these candidates, we selected eight mAbs (PODOC1 through PODOC8) with favorable selectivity profiles based on flow cytometry screening of tumor cell lines known to highly express podocalyxin (MDA-MB-231, CAOV-3, A-172), tumor cell lines known to express low levels of podocalyxin (MCF7, T47D, OVCAR-10) and non-tumor-derived human cells known to express podocalyxin (human umbilical vascular endothelial cells and HEK293 cells) (Table 1). Although we generated several antipodocalyxin mAbs with affinity for podocalyxin expressed on MDA-MB-231 cells, none of these exhibited an effect on tumor cell growth in monolayers or tumorsphere formation *in vitro* (data not shown).

**Figure 5:**
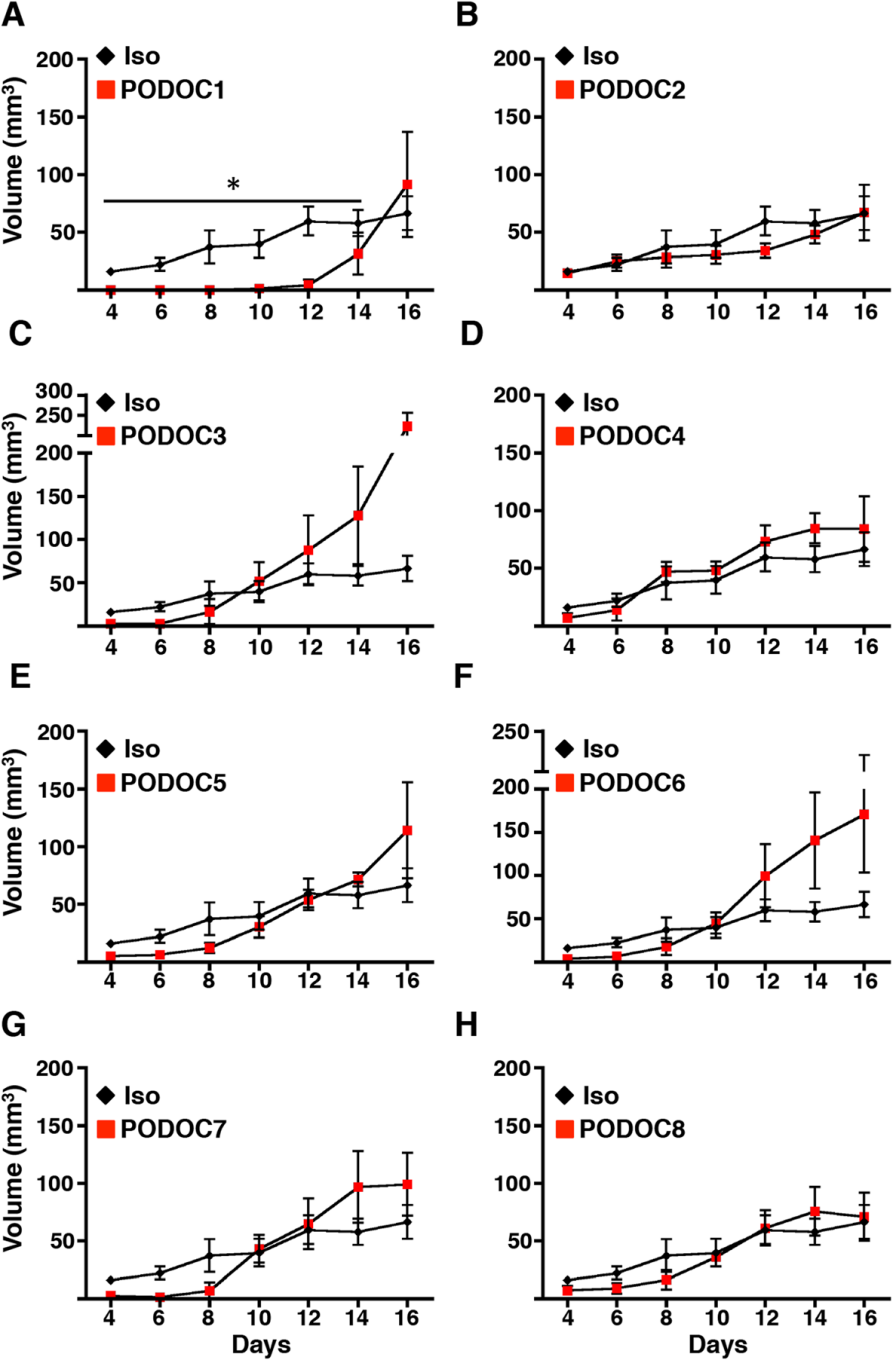
**Pretreatment of MDA-MB-231 cells with PODOC1 delays primary tumor development.** Growth curves from days 4 to 16 (post-injection) of tumors resulting from subcutaneously injected MDA-MB-231 cells pretreated with isotype control (Iso; red) or one of eight novel candidate antipodocalyxin antibodies (PODOC). **(A)** PODOC1 Ab (*p < 0.05; n = 3). **(B)** through **(H)** Growth curves for other candidate PODOCs (PODOC2 through PODOC8; n = 3). The data shown are representative of two independent experiments.

**Figure 6:**
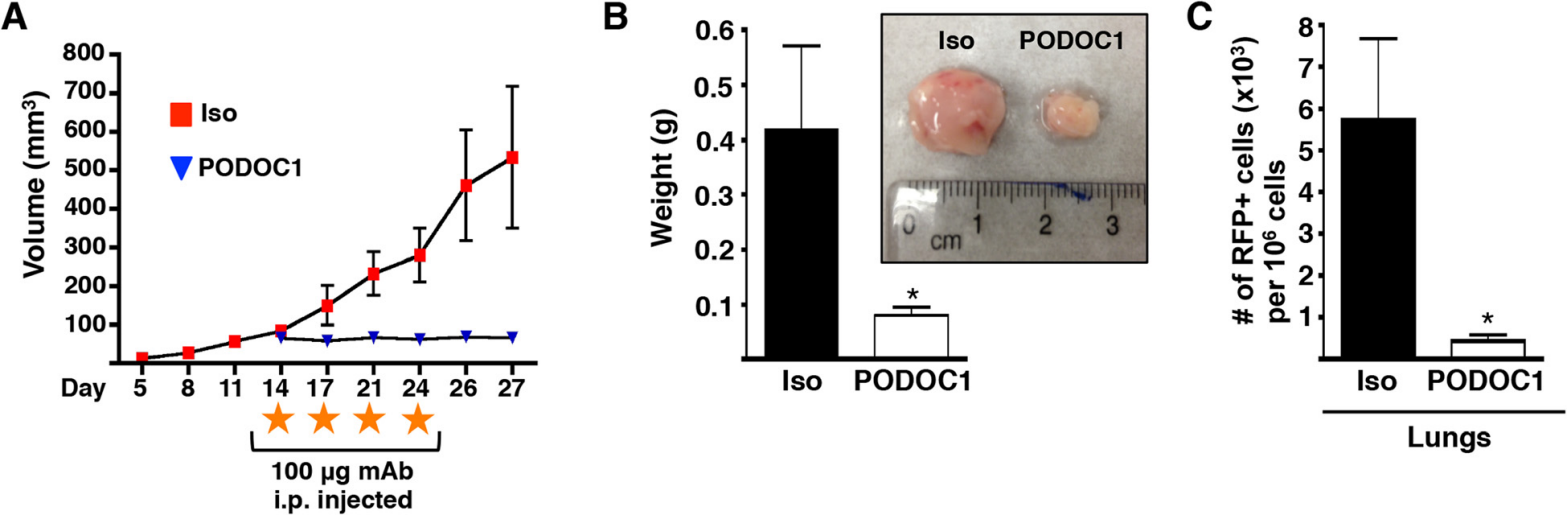
**Systemic treatment with antipodocalyxin antibody PODOC1 inhibits primary tumor development and metastasis to the lung. (A)** Growth curve of tumors from mice treated intraperitoneally (i.p.) with 100 μg of isotype control (Iso) or antipodocalyxin monoclonal antibody (mAb) PODOC1 on day 14 and every 3 to 4 thereafter days until being killed on day 27 (n = 5; *P < 0.05 by two-way analysis of variance) (right). Orange stars indicate i.p. administration of antibody. **(B)** Weight (g) of tumors treated with either Iso or PODOC1. Representative photograph shows tumors treated with either Iso or PODOC1 (inset) (n = 5; *P = 0.05). **(C)** Number of red fluorescent protein (RFP)-positive tumor cells per 10^6^ lung cells of mice with tumors treated i.p. with either Iso or PODOC1 as detected by flow cytometry (n = 5; *P < 0.05). The data shown are representative of two independent experiments.

Nevertheless, because podocalyxin expression appears to predominantly affect the ability of tumor cells to colonize tissues *in vivo* (rather than influencing their behavior *in vitro*), we went on to evaluate the effects of these antibodies in xenograft assays. Candidate mAbs were incubated with tumor cells immediately before s.c. injection into the flanks of recipient mice (pretreatment screen). One antipodocalyxin mAb (PODOC1) inhibited MDA-MB-231 growth and dissemination for 11 days (Figure 5A), whereas the Iso and 7 other podocalyxin-binding candidate mAbs (PODOC2 through PODOC8) failed to significantly alter tumor progression (Figure 5, B–H). Next, we assessed the ability of PODOC1 to inhibit established tumor growth (Figure 6). Cohorts of mice were treated therapeutically with PODOC1 or control Ab beginning 14 days after tumor injection, when a palpable tumor had already formed (s.c.). We consistently found that systemic treatment of tumor-bearing mice with PODOC1 completely inhibited tumor growth (Figure 6A, B) and, importantly, attenuated micrometastases to the lung (Figure 6C). Thus, we validated podocalyxin as a critical facilitator of tumor growth and progression and a therapeutic target for treatment in a preclinical model. To confirm that PODOC1 specifically binds to podocalyxin, Western blot analysis was performed on shCTRL and shPODXL MDA-MB-231 cell lysates. PODOC1 detected high levels of podocalyxin protein in shCTRL cells and significantly less in shPODXL cells (Additional file 6A). Additionally, in flow cytometric analyses, PODOC1 detected extracellular podocalyxin on shCTRL and lower levels on shPODXL MDA-MB-231 cells (Additional file 6B). It was also important to determine whether PODOC1 was specific for podocalyxin and did not bind to the closely related family member, CD34. Utilizing CHO cells transfected with human podocalyxin or CD34 (hCD34), PODOC1 was found to specifically detect overexpression of podocalyxin (Additional file 7B), whereas it failed to bind to hCD34 (Additional file 7C) or mock-transfected CHO cells (Additional file 7A). Thus, our data would support the argument that the PODOC1 Ab is highly specific and does not cross-react with a closely related sialomucin. We conclude that antibodies targeting the appropriate epitope on human podocalyxin can provide therapeutic benefit *in vivo*.

Finally, to test the potential efficacy of PODOC1 on late-stage metastatic disease, mice were injected s.c. with shCTRL^GFP^ MDA-MB-231 cells and tumors were allowed to reach a size larger than 500 mm^3^ prior to PODOC1 therapy. At this size, we find that metastatic lesions readily develop in the lungs. PODOC1 or Iso Ab was then administered to the mice with established tumor burdens at days 20, 26, 29 and 32 (Additional file 8A). Systemic treatment with PODOC1 appeared to marginally slow the growth of the primary tumor, although the difference was not statistically significant (Additional file 8A, B). However, PODOC1 treatment resulted in a dramatic reduction in the number of tumor nodules observed on the lung surface (Additional file 8C) recovered in total lung homogenates (Additional file 8D). We conclude that PODOC1 provides a clear therapeutic benefit, even in late-stage metastatic disease (that is, when metastatic organs are already colonized with tumor cells).

## Discussion

Although podocalyxin is expressed by a minor subset of primary breast tumors, these have been shown to be the most aggressive and difficult-to-treat breast cancers [10]. Importantly, podocalyxin expression is also a predictor of poor prognosis in many other cancers [10-15,17,18,20,31,32]. For example, patients with podocalyxin-positive colorectal carcinoma (where podocalyxin-expressing cells are often located at the invasive front of the primary tumor) have a higher probability of lymph node and distant metastases [31]. In addition, roughly 20% of stage III colorectal carcinomas express high levels of podocalyxin, and these represent a cohort that significantly benefits from adjuvant chemotherapy [31]. Comparatively, similar patients with low levels of tumor podocalyxin did not appear to significantly benefit from chemotherapy [31]. Knowing the likelihood of success before accepting a treatment that is difficult for some patients to tolerate has obvious decision-making benefit. Thus, podocalyxin-based “theranostic” and therapeutic strategies may prove to have broad applications if podocalyxin promotes primary tumor growth and metastasis in colorectal carcinoma and other epithelial cancers. It is now important to further explore the therapeutic efficacy of PODOC1 and similar reagents in a clinical setting. Because podocalyxin is present on normal human cells, including the vascular endothelium and kidney podocytes, extensive toxicologic studies will be needed to ensure the safety of a therapeutic Ab. However, we predict that because the podocalyxin-rich podocytes of the kidney are behind the blood filtration barrier in the urinary space, they may be spared exposure to PODOC1 therapy. Additionally, we have not observed any adverse effect of antibodies targeting mouse podocalyxin when systemically administered to wild-type mice. Furthermore, we have found that selective deletion of *Podxl* from mouse endothelia is well tolerated and nontoxic in mice [33]. Thus, the data would support the argument that interfering with podocalyxin expression on endothelia or binding of podocalyxin-reactive antibodies to the vasculature is unlikely to be toxic.

By identifying a requirement for podocalyxin in tumorigenesis, we can now begin to characterize the key molecular mechanisms by which podocalyxin promotes tumor cell growth and colonization of supportive niches. As is highlighted by Ki-67 staining of subcutaneous tumors, one function of podocalyxin may be the promotion of primary tumor cell proliferation *in vivo*. Intriguingly, this effect was observed only *in vivo* because loss of podocalyxin had no effect on the proliferation of cultured tumor cells. Notably, although silencing podocalyxin is detrimental to tumorsphere-forming efficiency of MDA-MB-231 cells, the PODOC1 mAb does not appear to alter tumorsphere-forming efficiency or proliferation *in vitro* (not shown). Tumor formation and metastasis *in vivo* are dependent on a number of cellular characteristics that are difficult to mimic *in vitro,* including migration to and colonization of a supportive niches, immune cell evasion, and survival of a hypoxic environment until the establishment of an adequate blood supply. It is likely that podocalyxin functions in these settings are multifactorial, and, thus far, we have been compelled to use *in vivo* models for these studies. It is now important to evaluate the molecular pathways podocalyxin impinges on *in vivo* that lead to altered tumor cell proliferation.

With regard to the early stages of tumor colonization of tissues, it is intriguing that the bulk of our shPODXL cells begin to reexpress *PODXL* during the first 7 to 14 days *in vivo.* Thus, our data would support an argument for an important influence of podocalyxin on an early tumor-initiating subset of cells. This notion is supported by the fact that, in our experimental lung metastasis assays, we found that silencing podocalyxin expression decreased the frequency (but not the size) of tumor nodules we observed. In aggregate, these data suggest that even transient depletion of podocalyxin expression during the early phase of tumor establishment can have a profound effect on late-stage growth of metastases, perhaps through impaired function or decreased frequency of a population of cells with TIC-like properties. We do not yet know which properties of TICs are influenced by podocalyxin expression, but they could include properties that enable tumor cells to proliferate or survive within a metastatic niche, including invasion, migration, adhesion and recruitment of supportive vasculature. It is noteworthy that podocalyxin, and its close relative CD34, are well-known markers of various subsets of stem cells during development and play a role in cell and tissue morphogenesis and colonization of developing tissues [7,9,26,27,31]. Likewise, podocalyxin was recently detected in an undifferentiated stemlike population in glioblastoma multiforme [11], and it is a well-known marker of both embryonic stem cells and embryocarcinomas [3,6]. Thus, in both normal development and neoplastic disease, podocalyxin expression has been linked to stem cell activity. Impaired tumor initiation would be consistent with known roles for podocalyxin and CD34-type proteins in blocking cell adhesion and facilitating chemokine-dependent inflammatory trafficking and hematopoietic stem cell engraftment of the bone marrow niche [34-36]. Importantly, in contrast to the wide variety of drugs that target tumor proliferation, there is a paucity of therapeutics that target TIC activity, and therefore the Ab strategy described here may be an important additional therapeutic avenue.

In many ways, our findings are complementary to those described in a recent publication by Lin *et al*. [37]. These authors provided provocative evidence that both podocalyxin and cortactin are important for the morphogenesis, motility, gelatin invasion and *in vivo* metastatic potential of MDA-MB-231 cells and showed that these proteins associate *in vitro*. Although they did not show that podocalyxin is essential for cortactin-mediated metastasis *in vivo,* these data do offer a potential mechanistic insight into podocalyxin function through a cortactin-containing complex. Given that our present study shows podocalyxin to be functionally important for tumorsphere-forming cells *in vitro* and the early phases of tumor colonization by a subset of cells *in vivo*, it is now important to validate the functional significance of the cortactin and podocalyxin interaction in this rare, but clinically critical, subset of tumor cells.

## Conclusions

It has previously been shown that podocalyxin expression in invasive breast carcinoma correlates with poor patient survival and that podocalyxin enhances the motility and invasiveness of breast cancer cell lines *in vitro* [10,19-21]. Here, using *in vivo* models of breast tumor growth and metastasis, we show that podocalyxin has a causal role in promoting the growth and proliferation of solid tumors and enhancing the metastasis of tumor cells to distant organs. We found that silencing podocalyxin expression in MDA-MB-231 cells, an aggressive triple-negative, basal-like breast cancer cell line, severely impaired primary tumor growth and metastasis to the lung, liver and bone marrow in a xenograft model. We corroborated these results in a syngeneic mouse model using fully immunocompetent mice by silencing podocalyxin expression in mouse mammary tumor 4T1 cells. Thus, in both mouse and human breast tumor cells, podocalyxin plays a critical role in disease progression. Furthermore, we have developed a unique mAb that targets podocalyxin and, in preclinical mouse studies, inhibits tumor growth and metastatic progression.

## Additional files

Additional file 1:
**Podocalyxin expression can be efficiently knocked down in MDA-MB-231 cells.** shCTRL and shPODXL MDA-MB-231 whole-cell lysates (5 × 10^4^ cells) were resolved by SDS-PAGE and analyzed by Western blotting using antipodocalyxin clone 3D3 (1:4,000; Santa Cruz Biotechnologies, Santa Cruz, CA, USA). An antibody against β-actin (1:10,000; Sigma-Aldrich) was used as a loading control. Additional file 2:
**Proliferation is not affected by podocalyxin expression in MDA-MB-231 cells in monolayer culture.** A 3-(4,5-dimethylthiazol-2-yl)-5-(3-carboxymethoxyphenyl)-2-(4-sulfophenyl)-2H-tetrazolium proliferation assay was performed on shCTRL and shPODXL MDA-MB-231 cells 48, 72 and 96 hours after initial seeding. Proliferation was quantified by the amount of formazan product detected at 490-nm absorbance using a microplate reader. The level of proliferation of shCTRL was compared with shPODXL cells over time (nonsignificant by two-way analysis of variance). All values are graphed as mean ± SEM. Additional file 3:
**shCTRL and shPODXL cells form tumorspheres of similar size and morphology.** A total of 5 × 10^3^ shCTRL or shPODXL MDA-MB-231 cells were cultured for 7 days in MammoCult medium. **(A)** Tumorsphere size was calculated using ImageJ software (pixels). **(B)** Representative images of shCTRL and shPODXL MDA-MB-231 tumorspheres. **(C)** Murine podocalyxin expression can be overexpressed in human MCF7 breast tumor cells. MCF7^pIRES^ control and MCF7^Podxl^ lysates were resolved by SDS-PAGE and analyzed by Western blotting using a goat anti-mouse podocalyxin antibody (1 μg/ml; R&D Systems). An antibody against β-actin was used as a loading control. **(D)** Flow cytometry was performed to detect the level of extracellular murine podocalyxin on MCF7^pIRES^ (left; blue) and MCF7^Podxl^ cells (right; red). A goat anti-mouse podocalyxin antibody (2.5 μg/10^6^ cells; R&D Systems) and an isotype against normal goat IgG (gray) were used. Additional file 4:
**Podocalyxin is reexpressed in primary MDA-MB-231 tumors after 14 days** ***in vivo*** **.** A total of 1 × 10^6^ shCTRL^GFP^ or shPODXL^GFP^ MDA-MB-231 cells were injected s.c. into the right and left flanks of NSG mice. After 14 days, mice were killed and perfused with 10 ml of ice-cold PBS, and tumors were excised. **(A)** One-third of each shCTRL and shPODXL tumor was processed for flow cytometric analysis using a 2-U/ml collagenase solution for 1 hour at 37°C and stained for extracellular podocalyxin using one of two antibodies (PODOC1 or goat antihuman podocalyxin; R&D Systems). The upper two histograms display the level of surface podocalyxin expression in shCTRL^GFP^ cells (left; blue) compared with shPODXL^GFP^ cells (right, blue) as detected by PODOC1 antibody. A goat antihuman AF647 (2 μg/ml; Invitrogen) secondary-only control is shown in red. The lower two histograms display the level of surface podocalyxin expression in shCTRL^GFP^ cells (left; blue) compared with shPODXL^GFP^ cells (right; blue) as detected by goat antihuman podocalyxin (2 μg/ml; R&D Systems) followed by chicken anti-goat AF647 (Invitrogen). Normal goat IgG isotype control is shown in red. **(B)** One-third of the tumors were processed “fresh” for Western blot analysis by directly homogenizing them in 500 μl of radioimmunoprecipitation assay (RIPA) lysis buffer, and the final third of the tumors were freeze-thawed (F/T) by snap-freezing in dry ice and storing them at −80°C for 1 hour. F/T tumors were homogenized in 500 μl of RIPA lysis buffer. A BCA assay was performed, and equal amounts of protein were resolved by SDS-PAGE and analyzed by Western blotting using antipodocalyxin clone 3D3 (1:4,000; Santa Cruz Biotechnologies). An antibody against β-actin (1:10,000; Sigma-Aldrich) was used as a loading control. Additional file 5:
**Metastatic lung nodules resulting from MDA-MB-231 cells or 4D1 cells are more prevalent in number, but not of greater size when compared with their respective podocalyxin knockdown lines. (A)** NSG mice were injected (i.v.) with a 50:50 mixture of shCTRL^GFP^ and shPODXL^RFP^ MDA-MB-231 cells (5 × 10^4^ cells). After 6 weeks, mice were killed and their lungs and fluorescent nodules on the lungs were imaged using a fluorescence dissecting microscope. The GFP and RFP channels were merged as a composite image to show nodules arising from both shCTRL and shPODXL cells. **(B)** BALB/c mice were injected (i.v.) with 1 × 10^5^ vector control (VC) or shPodxl mouse murine 4D1 tumor cells. After 2 weeks, lungs were perfused with ice-cold PBS, fixed in 10% buffered formalin, embedded in paraffin and sectioned. Representative lung sections containing VC or shPodxl nodules were stained with H&E. Additional file 6:
**PODOC1 antibody specifically detects podocalyxin expression in MDA-MB-231 cells. (A)** shCTRL and shPODXL MDA-MB-231 whole-cell lysates were resolved by SDS-PAGE and analyzed by Western blotting using candidate therapeutic antibody PODOC1 (1 μg/ml). An antibody against β-actin was used as a loading control. **(B)** Podocalyxin expression on shCTRL^GFP^ (solid) and shPODXL^GFP^ (dashed) MDA-MB-231 cells relative to secondary-only control (shaded), as detected using PODOC1 (10 μg/ml) antibody followed by goat antihuman AF647 secondary antibody (2 μg/ml; Invitrogen) (right). Additional file 7:
**PODOC1 antibody specifically interacts with podocalyxin and does not bind to CD34.** Human CD34 (hCD34) and podocalyxin were transiently overexpressed in CHO cells. Flow cytometry was performed on CHO cells transfected with **(A)** human podocalyxin (CHO^PODXL^) or **(B)** human CD34 (CHO^hCD34^) or **(C)** mock sequence (CHO^Mock^) and stained with either PODOC1 (left) (10 μg/ml) or mouse antihuman CD34 fluorescein isothiocyanate–conjugated antibody (right) (1:50; Invitrogen). Additional file 8:
**Systemic treatment with PODOC1 inhibits metastasis to the lung in mice with large (>500 mm** ^**3**^ **) primary MDA-MB-231 tumors.** A total of 1 × 10^6^ shCTRL^GFP^ MDA-MB-231 cells were injected s.c. into the flank of NSG mice and allowed to develop into solid tumors over 20 days. **(A)** Growth curve of tumors from mice treated intraperitoneally (i.p.) with 4.5 mg/kg of isotype control (Iso) or PODOC1 Ab on day 20 and at three time points until the mice were killed on day 36 (nonsignificant (n.s.) by two-way analysis of variance; n = 5) (right). Orange stars indicate i.p. administration of antibody. **(B)** Weight (g) of tumors treated with either Iso or PODOC1 antibody (n = 5; n.s. by Student’s *t*-test). **(C)** Representative bright-field and fluorescence microscopic images of lungs showing shCTRL^GFP^ tumor nodules from mice that had been systemically treated with Iso (upper two panels) or PODOC1 (lower two panels). **(D)** Percentage of GFP-positive tumor cells in the lungs of mice with tumors treated i.p. with either isotype or PODOC1 antibody as detected by flow cytometry (n = 5; **P* < 0.05).

## Abbreviations

Ab: Antibody
AF: Alexa Fluor
CHO: Chinese hamster ovary cells
DMEM: Dulbecco’s Modified Eagle’s medium
EDTA: Ethylenediaminetetraacetic acid
ER: Estrogen receptor
FBS: Fetal bovine serum
F/T: Freeze-thawed
GFP: Green fluorescent protein
H&E: Hematoxylin and eosin
HEK293: Human embryonic kidney 293
HER2: Human epidermal growth factor receptor 2
HUVEC: Human umbilical vascular endothelial cell
IgG: Immunoglobulin G
i.p.: Intraperitoneal
Iso: Isotype
i.v.: Intravenous
mAb: Monoclonal antibody
MEP21: Myb-Ets progenitor 21
NSG: Nonobese diabetic severe combined immunodeficiency, interleukin 2 gamma chain deficiency, NOD.Cg-*Prkdc*^scid^*Il2rg*^*t*m1Wjl^/SzJ
PBS: Phosphate-buffered saline
PCLP1: Podocalyxin-like protein 1
PgR: Progesterone receptor
PODO: Podocalyxin
RFI: Relative fluorescence intensity
RFP: Red fluorescent protein
RIPA: Radioimmunoprecipitation assay
RLU: Relative light units
s.c.: Subcutaneous
SEM: Standard error of the mean
shCTRL: Scrambled short-hairpin RNA control
shPODXL: Short-hairpin RNA targeting *PODXL*
shRNA: Short-hairpin RNA
4T1-luc: Luciferase-expressing 4T1 cells
TIC: Tumor-initiating cell
VC: Vector control
