# Supplementary figures and images for: Podocalyxin enhances breast tumor growth and metastasis and is a target for monoclonal antibody therapy
Source: Breast Cancer Res. 2015 Mar 27;17(1):46. doi: 10.1186/s13058-015-0562-7 (PMC4423095; doi:10.1186/s13058-015-0562-7)

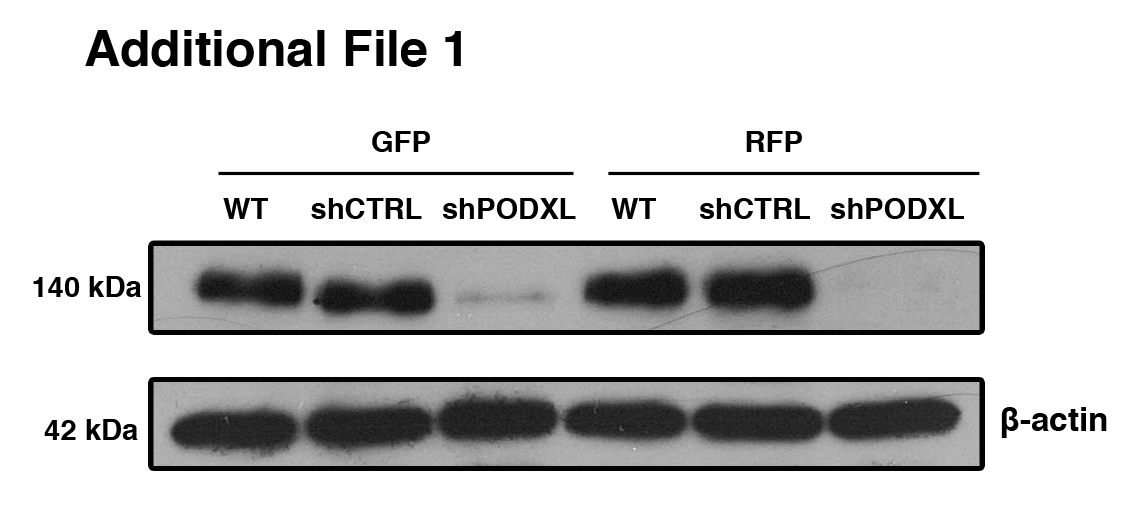

Supplement: Additional file 1: — Podocalyxin expression can be efficiently knocked down in MDA-MB-231 cells. shCTRL and shPODXL MDA-MB-231 whole-cell lysates (5 × 10^4^ cells) were resolved by SDS-PAGE and analyzed by Western blotting using antipodocalyxin clone 3D3 (1:4,000; Santa Cruz Biotechnologies, Santa Cruz, CA, USA). An antibody against β-actin (1:10,000; Sigma-Aldrich) was used as a loading control. [file 13058_2015_562_MOESM1_ESM.tiff]

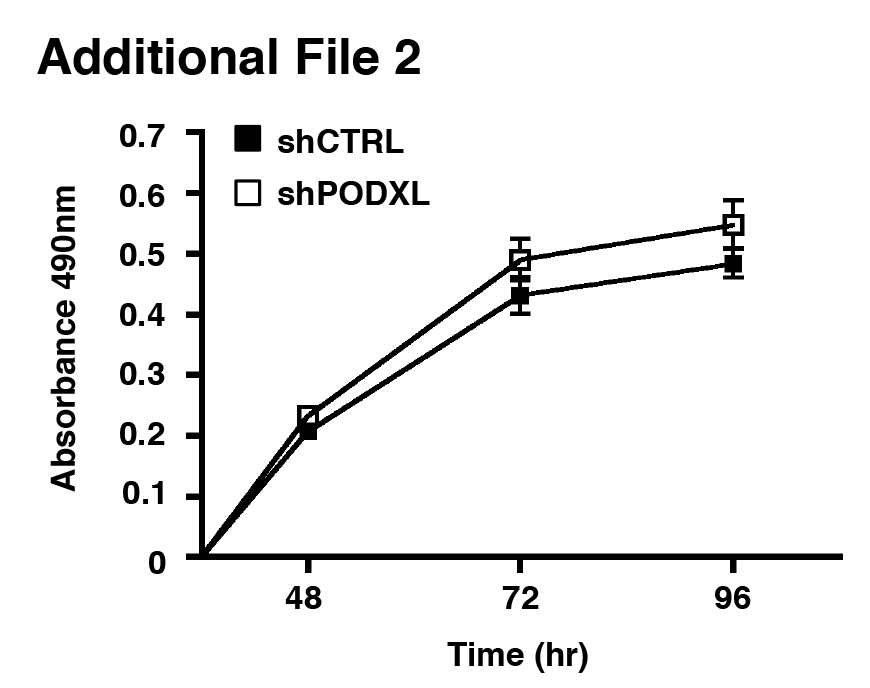

Supplement: Additional file 2: — Proliferation is not affected by podocalyxin expression in MDA-MB-231 cells in monolayer culture. A 3-(4,5-dimethylthiazol-2-yl)-5-(3-carboxymethoxyphenyl)-2-(4-sulfophenyl)-2H-tetrazolium proliferation assay was performed on shCTRL and shPODXL MDA-MB-231 cells 48, 72 and 96 hours after initial seeding. Proliferation was quantified by the amount of formazan product detected at 490-nm absorbance using a microplate reader. The level of proliferation of shCTRL was compared with shPODXL cells over time (nonsignificant by two-way analysis of variance). All values are graphed as mean ± SEM. [file 13058_2015_562_MOESM2_ESM.tiff]

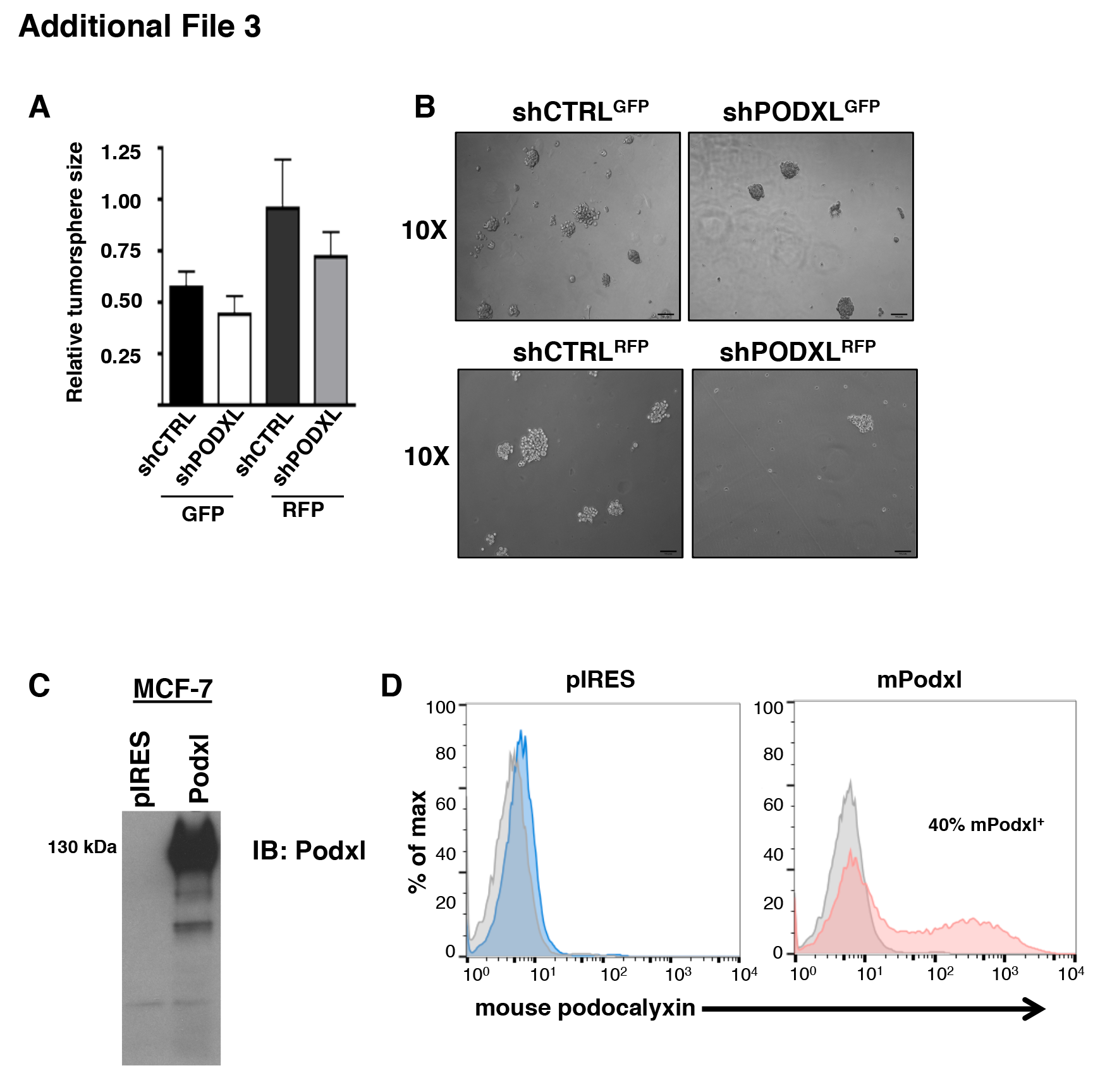

Supplement: Additional file 3: — shCTRL and shPODXL cells form tumorspheres of similar size and morphology. A total of 5 × 10^3^ shCTRL or shPODXL MDA-MB-231 cells were cultured for 7 days in MammoCult medium. (A) Tumorsphere size was calculated using ImageJ software (pixels). (B) Representative images of shCTRL and shPODXL MDA-MB-231 tumorspheres. (C) Murine podocalyxin expression can be overexpressed in human MCF7 breast tumor cells. MCF7pIRES control and MCF7Podxl lysates were resolved by SDS-PAGE and analyzed by Western blotting using a goat anti-mouse podocalyxin antibody (1 μg/ml; R&D Systems). An antibody against β-actin was used as a loading control. (D) Flow cytometry was performed to detect the level of extracellular murine podocalyxin on MCF7pIRES (left; blue) and MCF7Podxl cells (right; red). A goat anti-mouse podocalyxin antibody (2.5 μg/106 cells; R&D Systems) and an isotype against normal goat IgG (gray) were used. [file 13058_2015_562_MOESM3_ESM.tiff]

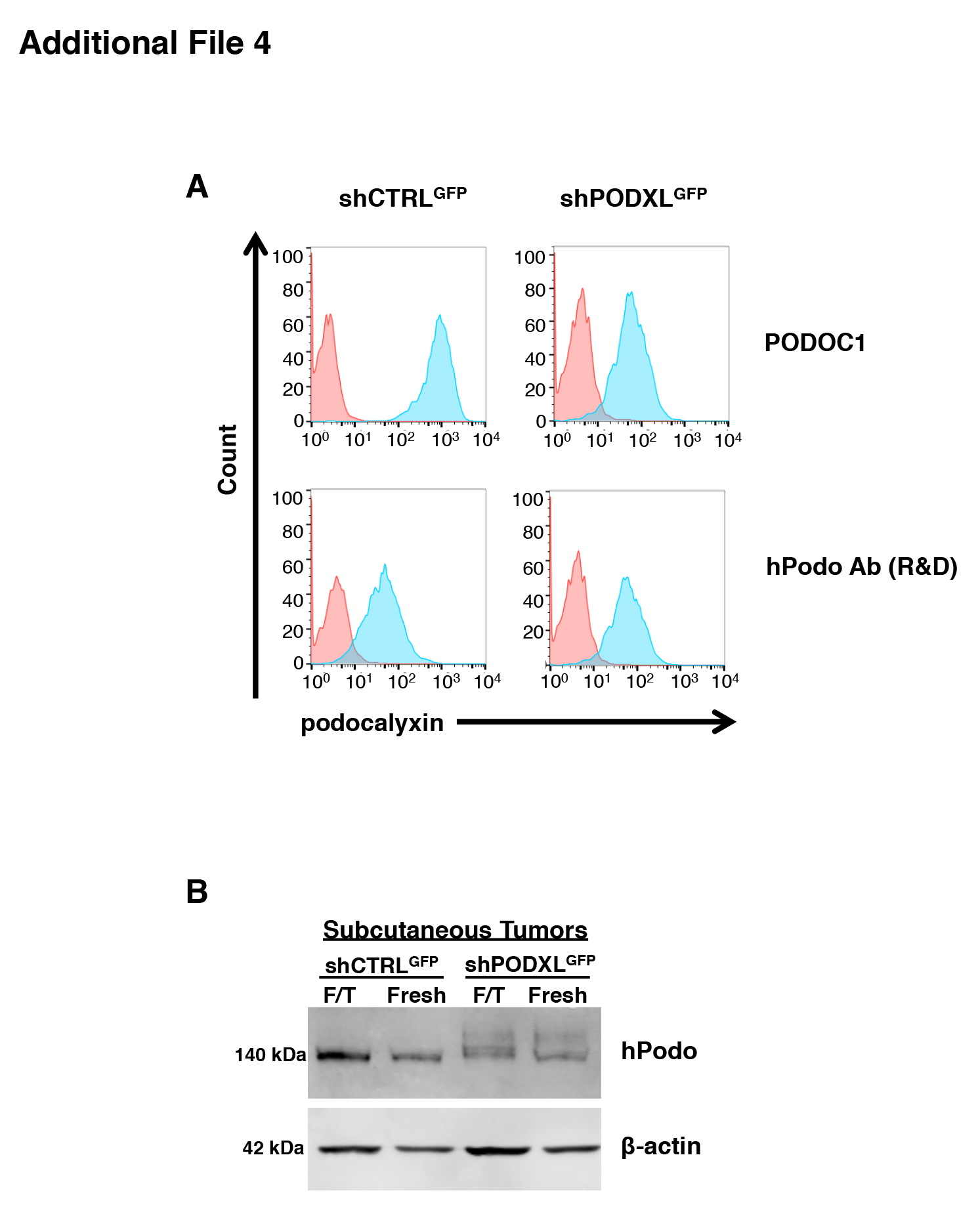

Supplement: Additional file 4: — Podocalyxin is reexpressed in primary MDA-MB-231 tumors after 14 days in vivo . A total of 1 × 10^6^ shCTRLGFP or shPODXLGFP MDA-MB-231 cells were injected s.c. into the right and left flanks of NSG mice. After 14 days, mice were killed and perfused with 10 ml of ice-cold PBS, and tumors were excised. (A) One-third of each shCTRL and shPODXL tumor was processed for flow cytometric analysis using a 2-U/ml collagenase solution for 1 hour at 37°C and stained for extracellular podocalyxin using one of two antibodies (PODOC1 or goat antihuman podocalyxin; R&D Systems). The upper two histograms display the level of surface podocalyxin expression in shCTRLGFP cells (left; blue) compared with shPODXLGFP cells (right, blue) as detected by PODOC1 antibody. A goat antihuman AF647 (2 μg/ml; Invitrogen) secondary-only control is shown in red. The lower two histograms display the level of surface podocalyxin expression in shCTRLGFP cells (left; blue) compared with shPODXLGFP cells (right; blue) as detected by goat antihuman podocalyxin (2 μg/ml; R&D Systems) followed by chicken anti-goat AF647 (Invitrogen). Normal goat IgG isotype control is shown in red. (B) One-third of the tumors were processed “fresh” for Western blot analysis by directly homogenizing them in 500 μl of radioimmunoprecipitation assay (RIPA) lysis buffer, and the final third of the tumors were freeze-thawed (F/T) by snap-freezing in dry ice and storing them at −80°C for 1 hour. F/T tumors were homogenized in 500 μl of RIPA lysis buffer. A BCA assay was performed, and equal amounts of protein were resolved by SDS-PAGE and analyzed by Western blotting using antipodocalyxin clone 3D3 (1:4,000; Santa Cruz Biotechnologies). An antibody against β-actin (1:10,000; Sigma-Aldrich) was used as a loading control. [file 13058_2015_562_MOESM4_ESM.tiff]

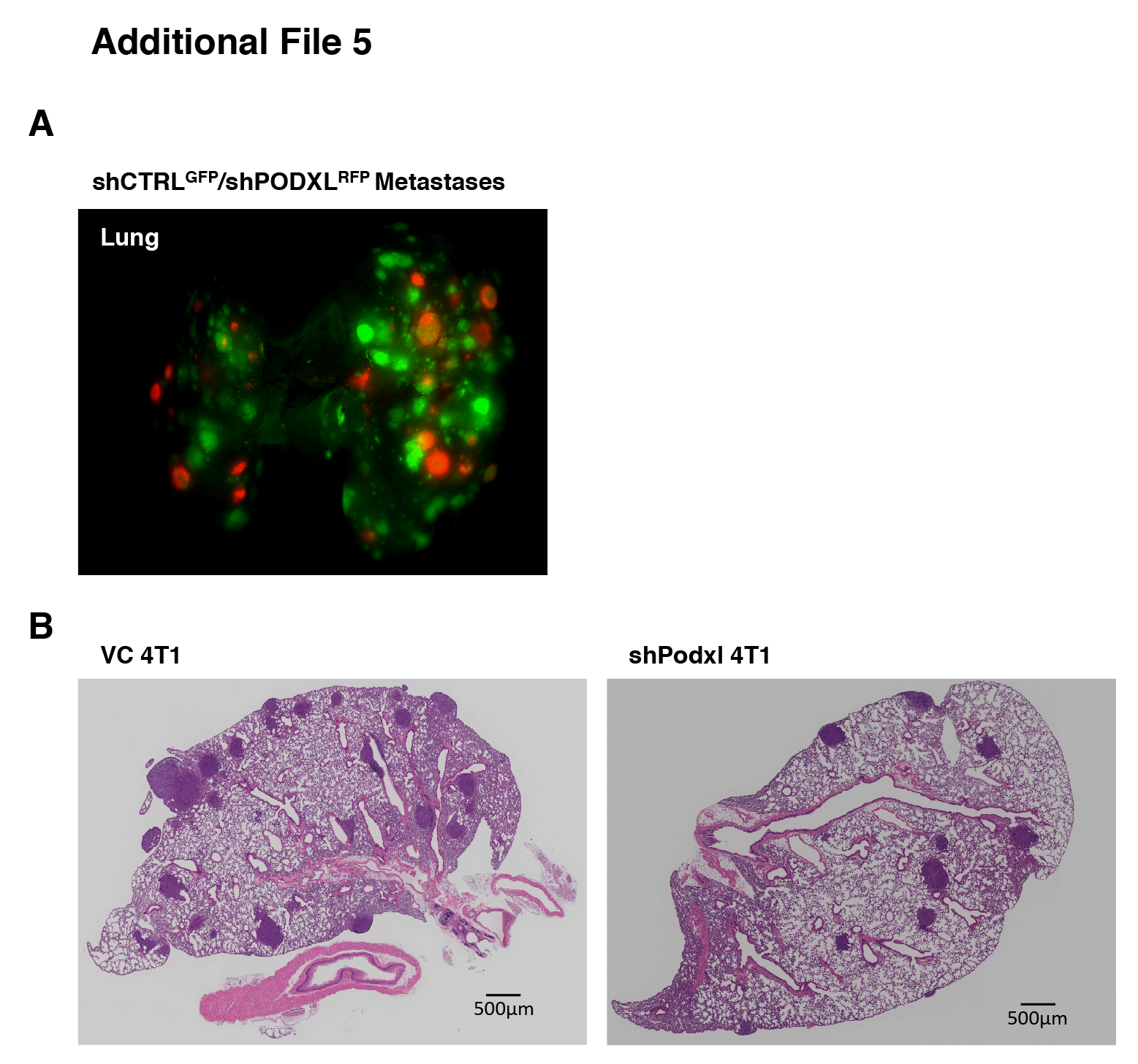

Supplement: Additional file 5: — Metastatic lung nodules resulting from MDA-MB-231 cells or 4D1 cells are more prevalent in number, but not of greater size when compared with their respective podocalyxin knockdown lines. (A) NSG mice were injected (i.v.) with a 50:50 mixture of shCTRLGFP and shPODXLRFP MDA-MB-231 cells (5 × 10^4^ cells). After 6 weeks, mice were killed and their lungs and fluorescent nodules on the lungs were imaged using a fluorescence dissecting microscope. The GFP and RFP channels were merged as a composite image to show nodules arising from both shCTRL and shPODXL cells. (B) BALB/c mice were injected (i.v.) with 1 × 10^5^ vector control (VC) or shPodxl mouse murine 4D1 tumor cells. After 2 weeks, lungs were perfused with ice-cold PBS, fixed in 10% buffered formalin, embedded in paraffin and sectioned. Representative lung sections containing VC or shPodxl nodules were stained with H&E. [file 13058_2015_562_MOESM5_ESM.tiff]

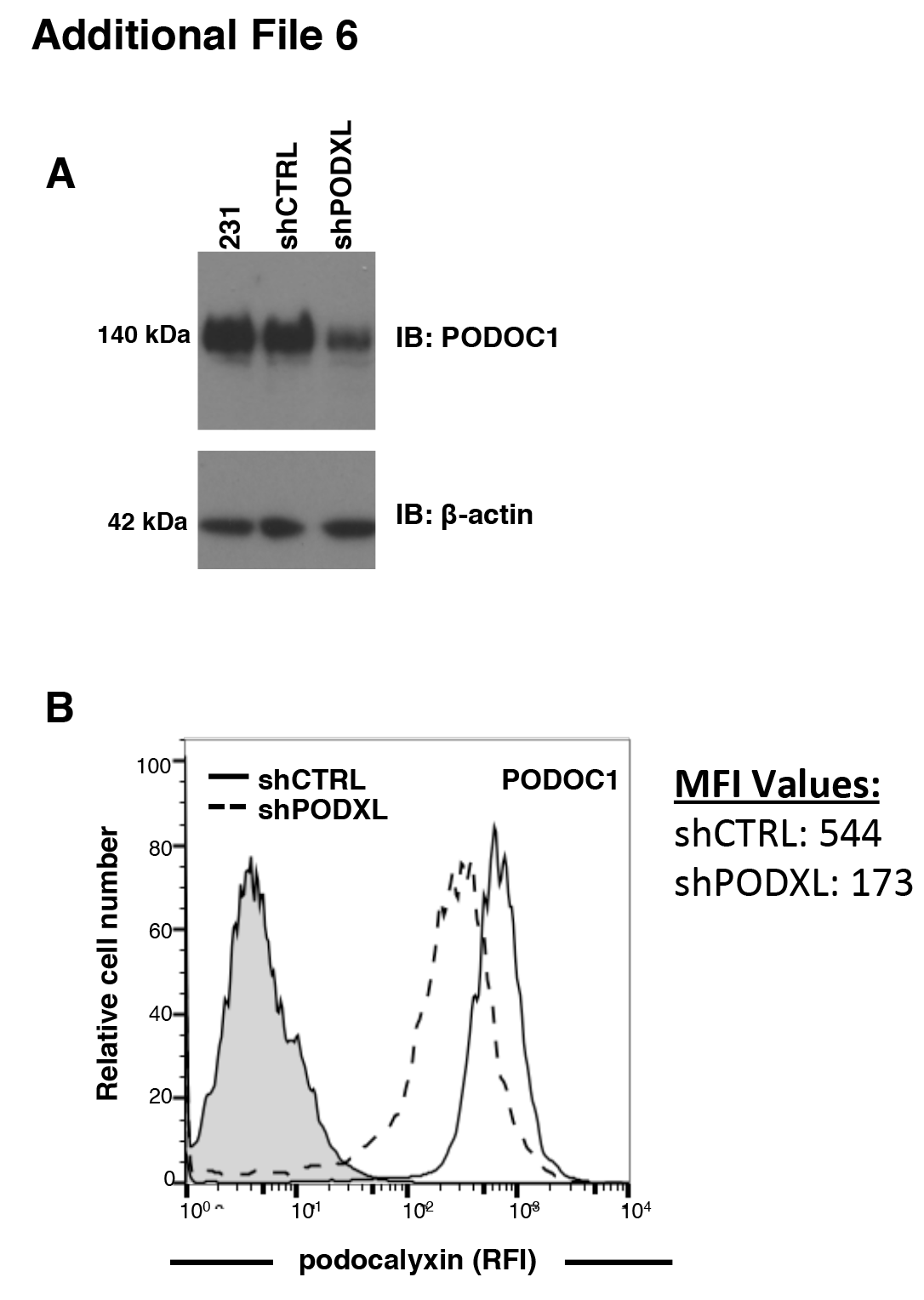

Supplement: Additional file 6: — PODOC1 antibody specifically detects podocalyxin expression in MDA-MB-231 cells. (A) shCTRL and shPODXL MDA-MB-231 whole-cell lysates were resolved by SDS-PAGE and analyzed by Western blotting using candidate therapeutic antibody PODOC1 (1 μg/ml). An antibody against β-actin was used as a loading control. (B) Podocalyxin expression on shCTRLGFP (solid) and shPODXLGFP (dashed) MDA-MB-231 cells relative to secondary-only control (shaded), as detected using PODOC1 (10 μg/ml) antibody followed by goat antihuman AF647 secondary antibody (2 μg/ml; Invitrogen) (right). [file 13058_2015_562_MOESM6_ESM.tiff]

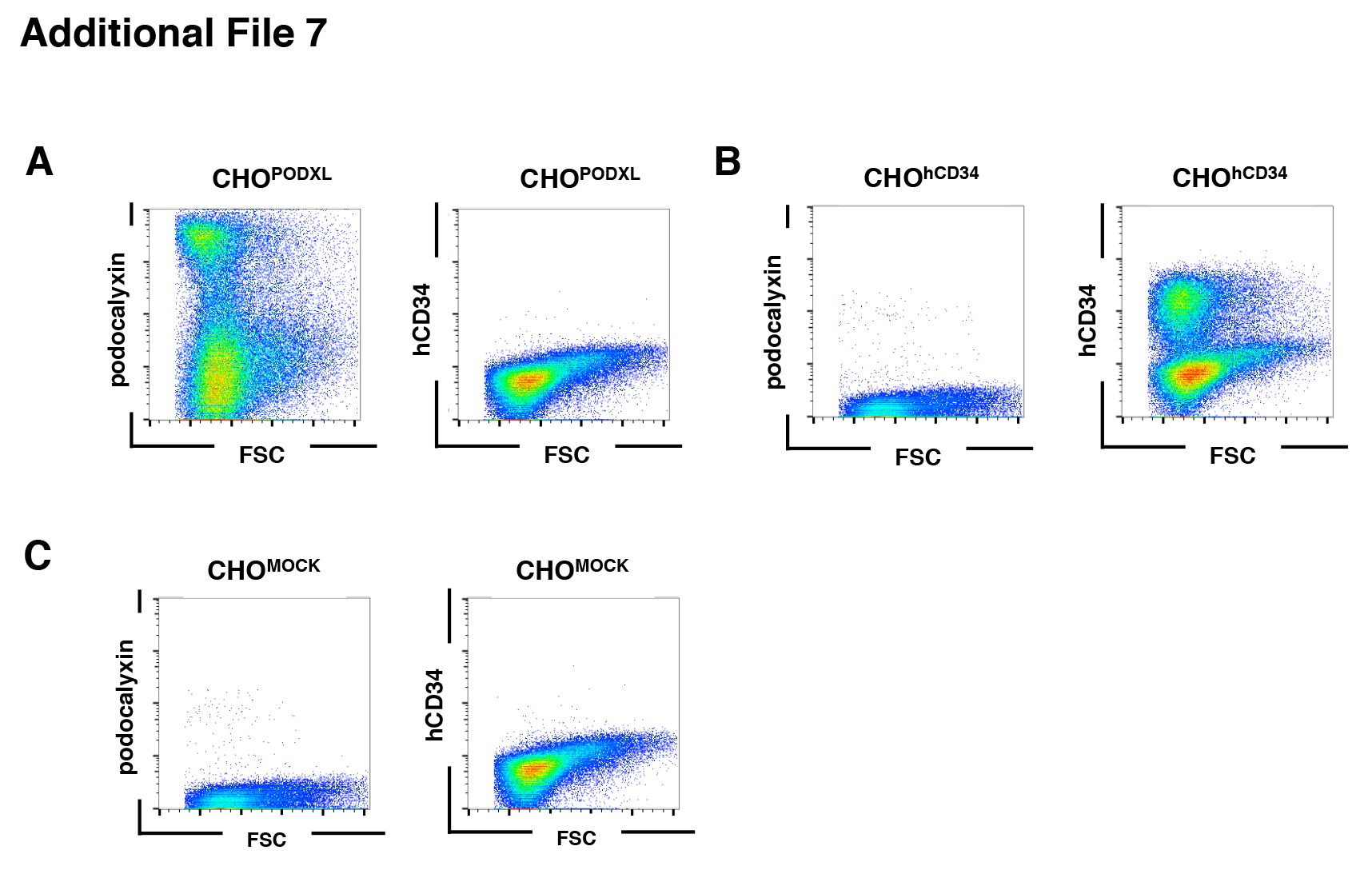

Supplement: Additional file 7: — PODOC1 antibody specifically interacts with podocalyxin and does not bind to CD34. Human CD34 (hCD34) and podocalyxin were transiently overexpressed in CHO cells. Flow cytometry was performed on CHO cells transfected with (A) human podocalyxin (CHOPODXL) or (B) human CD34 (CHOhCD34) or (C) mock sequence (CHOMock) and stained with either PODOC1 (left) (10 μg/ml) or mouse antihuman CD34 fluorescein isothiocyanate–conjugated antibody (right) (1:50; Invitrogen). [file 13058_2015_562_MOESM7_ESM.tiff]

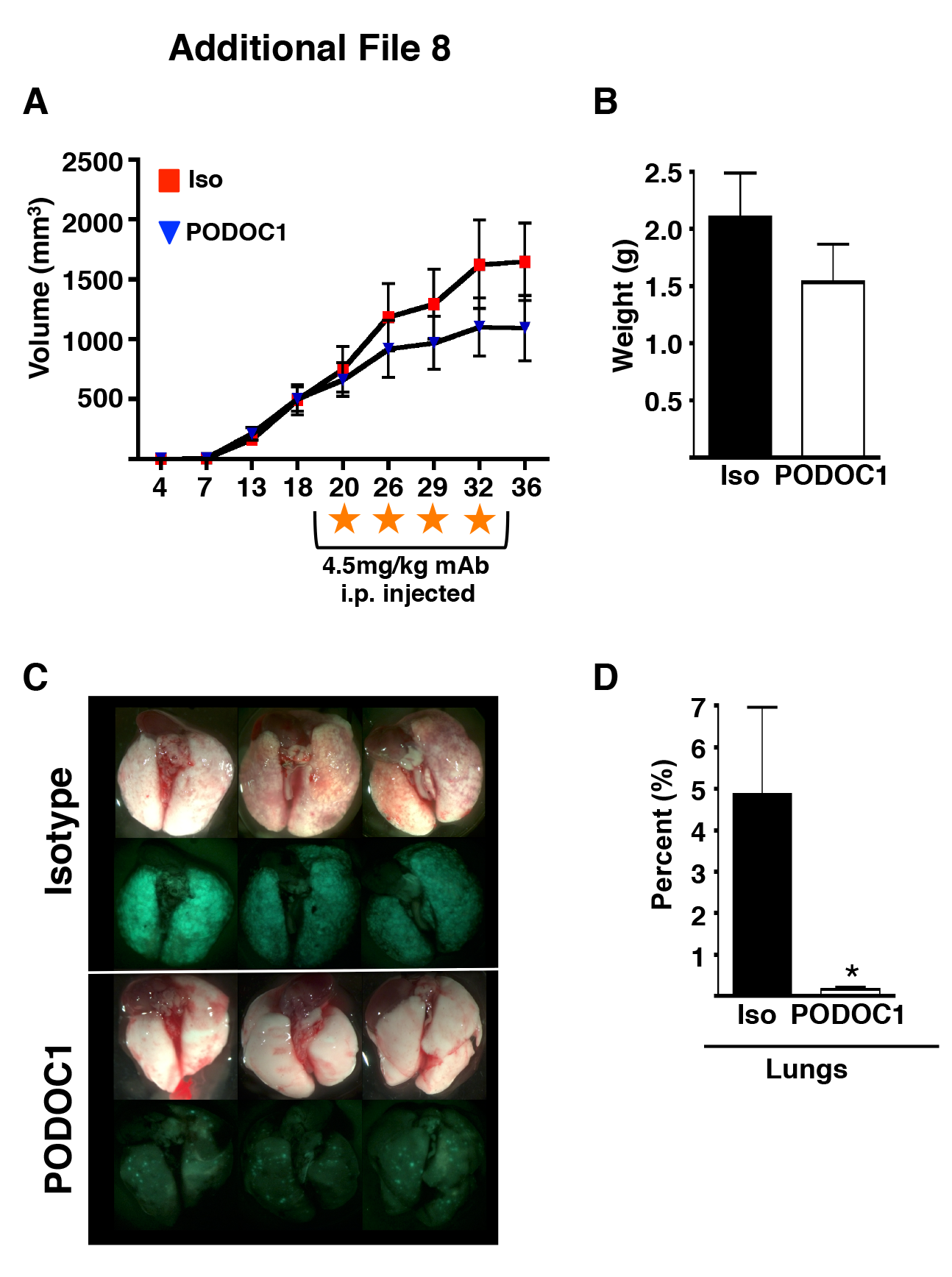

Supplement: Additional file 8: — Systemic treatment with PODOC1 inhibits metastasis to the lung in mice with large (>500 mm 3 ) primary MDA-MB-231 tumors. A total of 1 × 10^6^ shCTRLGFP MDA-MB-231 cells were injected s.c. into the flank of NSG mice and allowed to develop into solid tumors over 20 days. (A) Growth curve of tumors from mice treated intraperitoneally (i.p.) with 4.5 mg/kg of isotype control (Iso) or PODOC1 Ab on day 20 and at three time points until the mice were killed on day 36 (nonsignificant (n.s.) by two-way analysis of variance; n = 5) (right). Orange stars indicate i.p. administration of antibody. (B) Weight (g) of tumors treated with either Iso or PODOC1 antibody (n = 5; n.s. by Student’s t-test). (C) Representative bright-field and fluorescence microscopic images of lungs showing shCTRLGFP tumor nodules from mice that had been systemically treated with Iso (upper two panels) or PODOC1 (lower two panels). (D) Percentage of GFP-positive tumor cells in the lungs of mice with tumors treated i.p. with either isotype or PODOC1 antibody as detected by flow cytometry (n = 5; *P < 0.05). [file 13058_2015_562_MOESM8_ESM.tiff]
